# Optimizing biomedical information retrieval with a keyword frequency-driven prompt enhancement strategy

**DOI:** 10.1186/s12859-024-05902-7

**Published:** 2024-08-27

**Authors:** Wasim Aftab, Zivkos Apostolou, Karim Bouazoune, Tobias Straub

**Affiliations:** 1 Core Facility Bioinformatics, Biomedical Center, LMU Munich, Grosshaderner Str. 9, 82152 Martinsried, Germany; 2 Molecular Biology Division, Biomedical Center, LMU Munich, Grosshaderner Str. 9, 82152 Martinsried, Germany; 3 Department of Biochemistry and Molecular Biology, Pennsylvania State University, University Park, PA 16802 USA

**Keywords:** Biomedical question-answering, Prompt enhancement strategy, Large language models, Generative pretrained transformer

## Abstract

**Background:**

Mining the vast pool of biomedical literature to extract accurate responses and relevant references is challenging due to the domain's interdisciplinary nature, specialized jargon, and continuous evolution. Early natural language processing (NLP) approaches often led to incorrect answers as they failed to comprehend the nuances of natural language. However, transformer models have significantly advanced the field by enabling the creation of large language models (LLMs), enhancing question-answering (QA) tasks. Despite these advances, current LLM-based solutions for specialized domains like biology and biomedicine still struggle to generate up-to-date responses while avoiding “hallucination” or generating plausible but factually incorrect responses.

**Results:**

Our work focuses on enhancing prompts using a retrieval-augmented architecture to guide LLMs in generating meaningful responses for biomedical QA tasks. We evaluated two approaches: one relying on text embedding and vector similarity in a high-dimensional space, and our proposed method, which uses explicit signals in user queries to extract meaningful contexts. For robust evaluation, we tested these methods on 50 specific and challenging questions from diverse biomedical topics, comparing their performance against a baseline model, BM25. Retrieval performance of our method was significantly better than others, achieving a median Precision@10 of 0.95, which indicates the fraction of the top 10 retrieved chunks that are relevant. We used GPT-4, OpenAI's most advanced LLM to maximize the answer quality and manually accessed LLM-generated responses. Our method achieved a median answer quality score of 2.5, surpassing both the baseline model and the text embedding-based approach. We developed a QA bot, WeiseEule (https://github.com/wasimaftab/WeiseEule-LocalHost), which utilizes these methods for comparative analysis and also offers advanced features for review writing and identifying relevant articles for citation.

**Conclusions:**

Our findings highlight the importance of prompt enhancement methods that utilize explicit signals in user queries over traditional text embedding-based approaches to improve LLM-generated responses for specialized queries in specialized domains such as biology and biomedicine. By providing users complete control over the information fed into the LLM, our approach addresses some of the major drawbacks of existing web-based chatbots and LLM-based QA systems, including hallucinations and the generation of irrelevant or outdated responses.

**Supplementary Information:**

The online version contains supplementary material available at 10.1186/s12859-024-05902-7.

## Background

Retrieving precise answers and relevant references from large volumes of text is still a crucial but challenging task in biomedical research. The complexity of this endeavor arises from several characteristics and challenges inherent to biomedical literature. The language used is highly specialized and includes numerous acronyms, technical jargon, standardized nomenclature, and complicated medical terms. Moreover, the biomedical domain is ever evolving, featuring constant improvements and breakthroughs. Because of this rapid advancement, new information is constantly being added, making it difficult for retrieval systems to stay up to date. Furthermore, biomedical information is shared in various formats such as research papers, clinical trial reports, patent documents and so on. The structural complexities of each of these formats make it difficult to extract and interpret information with accuracy.

In addition to these challenges, biomedical literature uses synonyms, and highly context-dependent phrases which adds yet another level of complexity. Because language usage varies significantly, ambiguities may arise that make it challenging for information retrieval systems to understand the precise context or meaning intended in a query. Furthermore, biomedical research often intersects with other disciplines such as statistics, physics, and computer science etc. Therefore, to deliver thorough and pertinent information, retrieval systems must be able to integrate and understand concepts from these many domains.

Early NLP approaches which primarily used a mixture of rule-based algorithms, statistical analysis, and classical machine learning techniques made progress in solving these issues [1–4]. However, their ability to comprehend subtleties and context of complex biomedical texts was often limited, and they struggled to capture long-term dependencies in the text. This frequently resulted in inaccurate or irrelevant responses.

The emergence of transformer models has revolutionized the field of NLP. A much richer understanding of context and linguistic nuances has been made possible by Transformers, which process entire text sequences simultaneously. These models rely on utilizing deep learning and a novel use of self-attention mechanisms to process sequences of text [5]. Transformer-based models have demonstrated potential in capturing long-term dependencies in text, better interpreting context, and comprehending the nuances of natural language, resulting in improved accuracy and relevance in generated responses compared to earlier models [6–8].

The development of LLMs have further enhanced QA tasks, building upon the revolutionary developments initiated by early transformer models [6–9]. LLMs are instances of foundational models that are pre-trained on large amounts of unlabeled and self-supervised data, meaning the model learns from patterns in the data to produce generalizable and adaptable output. LLMs are applied particularly to text and text-like entities such as codes. These models can be tens of gigabytes in size and trained on enormous amounts of text. For instance, GPT-3 from OpenAI, is pre- trained on a corpus of 45 terabytes of text corpora from the web most likely encompassing scientific and biomedical literature [8]. In the context of biomedical QA, LLMs can generate answers to complex questions by leveraging their extensive pre-training and with an increase in the number of parameters, they typically demonstrate enhanced performance [8, 10]. These parameters essentially represent the size of an LLM's “brain,” and currently, there is a competitive trend in creating LLMs with bigger brains. Among many available LLMs, OpenAI's GPT-3 and 4 have gathered significant attention after their chatbot ChatGPT became widely recognized [11]. ChatGPT offers a browser interface for easy access to OpenAI’s LLMs, but these models can also be accessed via Application Programming Interfaces (APIs) for more customized applications such as querying a large pool of personalized information. However, training LLMs daily with newly added information is prohibitively expensive. As a result, a major issue with LLMs is that they may yield outdated responses due to not keeping up with latest literature. While OpenAI has integrated Bing search capabilities for ChatGPT plus subscribers to closely align responses with the currently available information on the web, this feature is presently limited to web-based access only, eventually limiting its applicability to nontrivial NLP tasks such as biomedical QA. Moreover, even with dynamic web access, ChatGPT plus users are limited to the initial search results as speed would become a bottleneck. In fact, for many complex queries, ChatGPT with Bing search takes longer time to generate answers. In addition, LLMs suffer from hallucination which in the current context refers to the phenomenon where the model generates responses that may sound plausible but are not factually correct.

Other web-based conversational search engines space include Perplexity.ai, which aims to generate answers using LLMs such as GPT-3.5, 4 and Claude, Mistral Large, and their in-house model which is currently experimental [12]. Though Perplexity.ai supports querying custom knowledge base via file uploads for premium users, it has restrictions on the number and size of the files. Therefore, the knowledge base of these chatbots that aim to cater all domains, are substantially limited to the information that is available freely on the web. This limitation can significantly affect the quality of responses from QA systems, especially in rapidly evolving fields like biology and biomedicine.

Researchers affiliated to institutes and universities typically have access to vast amounts of research content via institutional subscriptions. Additionally, they often obtain research papers through networks of fellow researchers in other universities, accumulating extensive personal libraries over time. Querying this huge pool of literature is beyond the capabilities of current web-based chatbots, indicating a gap in the applicability of these tools for thorough question answering.

More recently, researchers have proposed prompt engineering (PE) approaches for a variety of use cases to improve responses generated by an LLM [13–15]. A “prompt” refers to a textual input provided by the user to instruct and/or guide an LLM to generate meaningful responses. Prompt engineering is mainly about providing as much context as possible with providing few examples (few-shot learning) to enhance an LLM’s understanding of a given use-case. However, PE leverages pre-trained knowledge base wired into the parameter space of an LLM, which can lead to issues like outdated or fabricated responses mentioned earlier. Therefore, its effectiveness is somewhat limited in certain applications such as question answering (QA) especially within specialized domains like biomedicine. To address these issues, researchers have proposed prompt enhancement strategies (PESs) that utilize a retrieval-augmented generation (RAG) based architecture to dynamically extract contexts from a vast pool of information to augment user’s prompt [16]. This augmented/enhanced prompt then serves as the knowledge base for an LLM to seek answers.

In this manuscript, we focused on improving information retrieval for biomedical QA tasks by utilizing RAG-based PES. Within open domains QA tasks, PES based on retrieval-augmented architecture is a popular approach. It mainly relies on text embedding and similarity of vectors in a high dimensional space to extract contexts for prompts [17–19]. While this PES provided substantial progress toward addressing the aforementioned LLM-related issues, we found it fell short, particularly when dealing with specialized biomedical questions. For the biomedical domain, there are just very few RAG based frameworks such as Almanac and MedRAG which aim to create improved prompt for given queries [20, 21]. MedRAG is a medical QA toolkit that utilizes predefined corpora and for PubMed QA mainly comprising only abstracts from PubMed. However, for comprehensive QA, especially in closed domains like the biomedical one, the abstract information alone is often insufficient. Almanac, another QA framework, focuses on clinical medicine by dynamically extracting contexts through web searches. The relevant articles found are broken into smaller pieces and stored in a vector database to facilitate information retrieval. However, similar to ChatGPT with Bing search, user experience can suffer due to real time article searching and information retrieval approach adopted by Almanac. Additionally, Almanac requires curated clinical calculators to perform optimally, which demands effort and medical expertise from the user, making it more suitable for clinical/medical domain than general biomedical domain. Moreover, both the Almanac and MedRAG are command line tools which limits their accessibility for individuals with low computational skills.

To address these limitations, we developed an easy-to-use GUI framework called WeiseEule,^1^ where we adopt a prompt enhancement method based on retrieval-augmented architecture. Unlike retrieval methods using similarity of vectors, WeiseEule uses explicit signals from user queries to extract relevant contexts from the vast pool of biomedical literature. For comprehensive QA, our tool not only retrieves article abstracts but it also allows retrieving contexts from full texts. Furthermore, we offer article recommendation feature, which may aid in review or grant writing as well as focused reading. Additionally, our framework is open source and highly modular, providing advanced users the flexibility to add or remove features easily.

## Methods

**Fig. 1:**
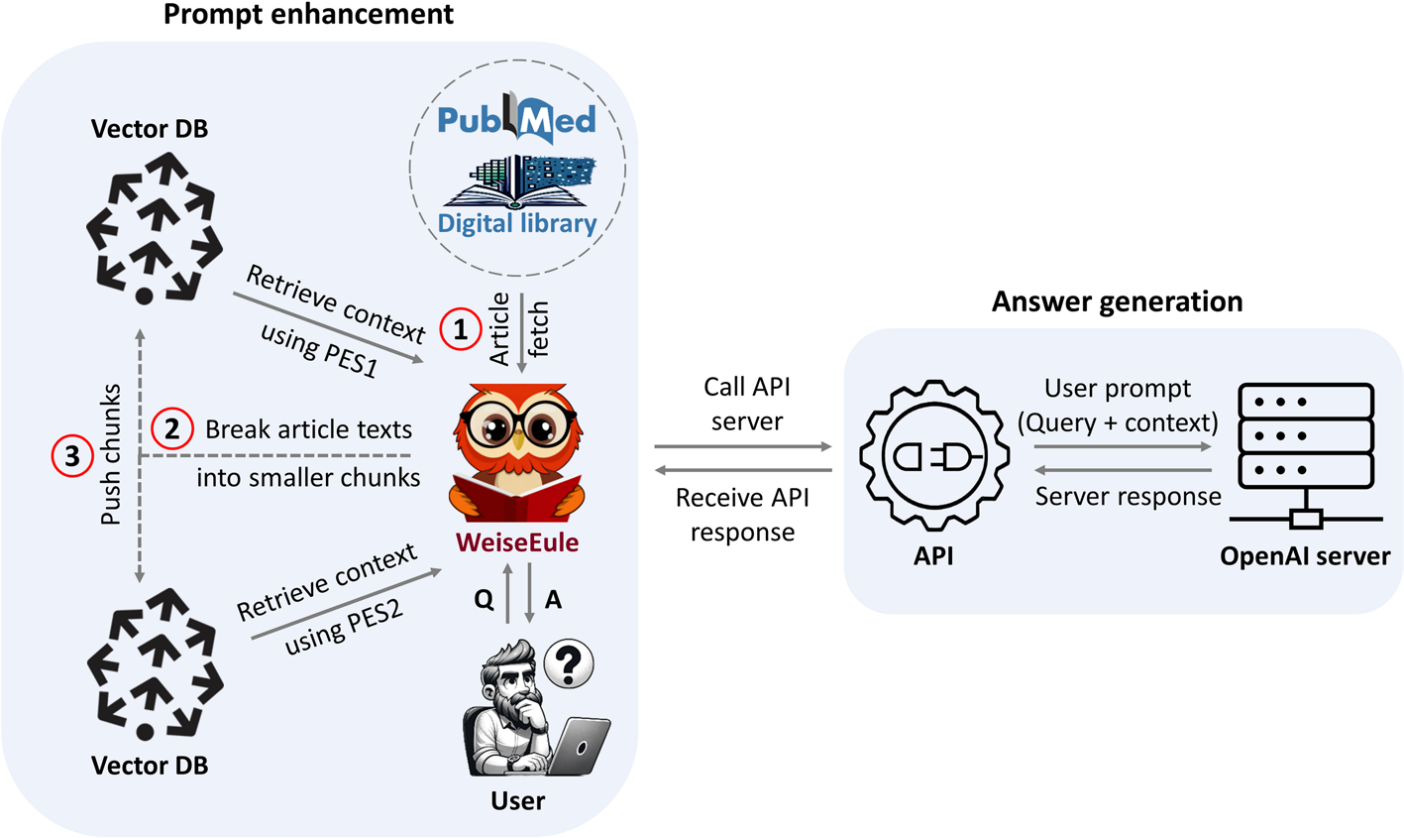
WeiseEule pipeline—The WeiseEule pipeline is designed to facilitate a seamless QA experience, combining namespace creation and the generative capacity of LLMs with strategic prompt design. Three necessary steps required to set up the knowledge base are indicated in red circles. Users can choose between PES1 and PES2 approaches to retrieve contexts

The conceptual underpinning of our algorithm is depicted in Fig. 1. The idea is to first create a knowledge base from research papers. Then using prompt enhancement techniques based on retrieval-augmented architecture (see sub section “Context retrieval based on vector similarity ranking (PES1)”, “Context retrieval based on keyword frequency ranking (PES2)”) construct relevant prompts that can be sent to LLM for generating answers to user questions. In the following sub sections, we describe the details of the prompt enhancement approaches.

### Knowledge base

**Fig. 2:**
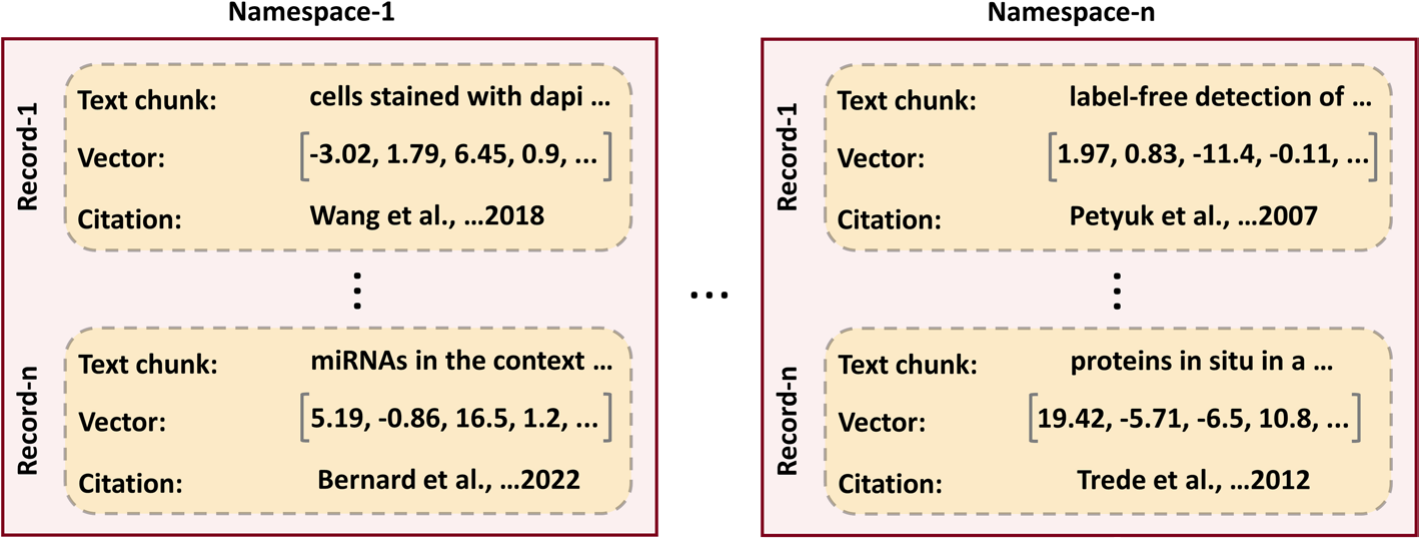
Namespacing. The concept of namespacing is foundational to our knowledge management approach. Within vector databases, namespaces serve as virtual containers that store relevant information. Each record in a namespace is a composite data structure that can hold a variety of metadata, such as the text chunk, its corresponding vector representation, and bibliographic references to its original source

A crucial step in our strategy is to generate a knowledge base (KB), which helps users to have total control of information that goes into the LLM and massively reduces the chances of hallucinations. To build a custom KB one can download articles from PubMed in text format or utilize already accumulated PDFs in a personal digital library. A critical task here is to break the article text into smaller pieces (chunks) so that they can fit into a prompt. Also, breaking down text into smaller chunks enable LLMs to focus on smaller, contextually meaningful units rather than complete documents, which can enhance performance. In WeiseEule, we employ *RecursiveCharacterTextSplitter* from LangChain, a package that uses a hierarchical structure to split text into more manageable, cohesive pieces [22]. It starts splitting at higher-level structures, like paragraphs and sentences and, then works recursively down to lower-level structures, such as words or characters, if needed. The goal of the splitter is to maintain readability and logical flow by keeping chunks below a user-defined maximum length. If initial splits exceed the size limit, the process keeps going at a finer granularity until the chunks fit the size requirements. This technique complies with size restrictions while maintaining the text's natural flow and meaning, making it suitable for further processing. Each of these chunks then need to be converted into sequence of numbers so that they can be used during text embedding-based PES (described below, Sections “Text to Numbers” and “Context Retrieval based on vector similarity ranking (PES1)”). Those vectors are subsequently pushed into a vector database (DB) for high performance access. Key to our KB creation is the concept of namespace (see Fig. 2). A Namespace serves as a container to hold relevant information in place. For example, if a user asks a query about *dosage compensation*, which refers to the mechanism organisms use to balance gene expression across different biological sexes, the answer will likely be in publications that list *dosage compensation* as a keyword. Therefore, if one accumulates all those research articles and put them in a virtual container, it then forms a namespace, provided it also receives a name tag, e.g., `dosage compensation articles`. Thus, a namespace with the chunks and vectors serves as a custom knowledge base for the prompt enhancement strategies discussed in this manuscript. For namespace generation, the WeiseEule app also downloads full texts, whenever they are available on the PubMed Central (PMC) server. For articles behind paywalls, WeiseEule offers namespace generation by allowing PDF uploads (See the sub-section ‘Namespace creation’ under the section ‘Salient features of the WeiseEule App’ under Results). As mentioned earlier, researchers at institutes and universities often have access to an extensive research content through institutional subscriptions and their networks of colleagues at other universities, which make it possible to obtain the PDFs for non-open access articles. Otherwise, the app only considers the abstracts for those articles. Next, the app saves the article records including PubMed ID (PMID), title, abstract, body text (when available) and citation information in a local SQLite DB. The local SQLite DB functions as a local literature database/store and monitors which article chunks are pushed into the cloud-based vector DB. This helps in preventing that records from the local DB are redundantly pushed into the vector DB, facilitating the maintenance of unique records in every namespace inside the vector DB.

In a vector DB, there can be multiple namespaces generated from different topics, the namespacing approach helps in organizing heterogeneous knowledge bases in a single vector DB or in one cloud account. However, for namespacing to function correctly, the next step is to convert the text chunks within a namespace into vectors as described below.

### Text to numbers

**Fig. 3:**
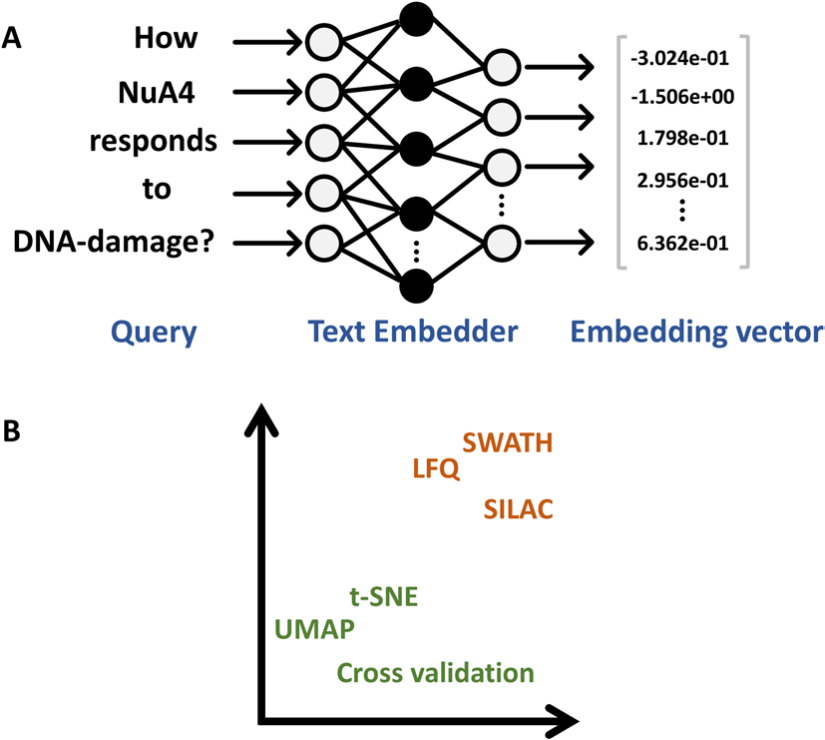
How an LLM understands texts—An LLM interprets texts by first turning them into embedding vectors. **A** The conversion aims to preserve the semantic meaning of the text by making the LLM to capture as much as possible the connections between various text parts. **B** Text embeddings are a type of representation that allows words with similar meanings to be represented similarly

The transformation of text into embedding vectors is called text embedding. There are specialized models, called *text embedders*, responsible for this [6, 23, 24]. This conversion retains the text's semantic meaning (Fig. 3A), ensuring the LLM captures relationships between different text parts. Text embeddings are a type of representation that allows words with similar meanings to be represented similarly (Fig. 3B). It is a projection of text into a high dimensional latent space resulting in compression of text into a list of floating-point numbers (vector). This is typically done by learning dense/compressed representation through context. In this study, we compared four different text embedders:

- Text-embedding-ada-002: It is an improved version of previous generations of embedding models such as text-search-davinci-*-001 and text-search-curie-*-001 etc. from OpenAI [25]. This model was trained on large amount of data from general domain texts such as Wikipedia [26].
- BioBERT-v1.1: This model was obtained after pre-training the Bidirectional Encoder Representations from Transformers (BERT) language model on large amount biomedical text [27].
- BioGPT: This model was obtained after pre-training Generative Pre-trained Transformer (GPT) model on massive number of abstracts and titles from PubMed articles [28]. The pre-training was performed on GPT-2 model architecture.
- MedCPT (Medical Contrastive Pre-trained Transformer): MedCPT is specifically designed for zero-shot biomedical information retrieval from PubMed [29]. It was trained on an extensive number of query-article pairs from PubMed logs.

Once the chunks are converted into vectors, they need to be stored in a vector DB for faster and efficient querying. The aim is to efficiently extract chunks relevant to construct high quality prompt for a given query. This approach forms the core of the prompt enhancement approach discussed in the next section.

### Context retrieval based on vector similarity ranking (PES1)

To store the vectors, we choose Pinecone DB because it provides ultra-low query latency even with large number of vectors [30] and offers a free tier which allow users to quickly prototype and test their applications. When a user asks a question, it is converted to a vector which is then searched in vector DB to extract chunks that have high similarity with the query vector. The cosine similarity ${S}_{\theta }(q, c)$ between a query vector (q) and the vector corresponding to some chunk (c) is given by Eq. (1)

1 $${S}_{\theta }(q, c)= \frac{\sum_{i=1}^{n}{q}_{i}.{c}_{i}}{\sqrt{\sum_{i=1}^{n}{q}_{i}^{2}} .\sqrt{\sum_{i=1}^{n}{c}_{i}^{2}}}$$

where the numerator computes the dot product between the two n-dimensional vectors q and c, while the denominator calculates the product of their Euclidean lengths [31]. The main reasons to use cosine similarity as a metric to compute similarity between vectors is as follows:

- Semantic similarity: It measures the cosine of the angle between the two embedding vectors which effectively captures the semantic similarity between them [32].
- Normalization: It considers the direction of the vectors rather than their magnitude by normalizing for the length of the vectors. This is especially helpful in text mining applications where the frequency (and thus the magnitude) of words can vary significantly, while it is the directional similarity that conveys meaningful semantic relationships [31].

**Fig. 4:**
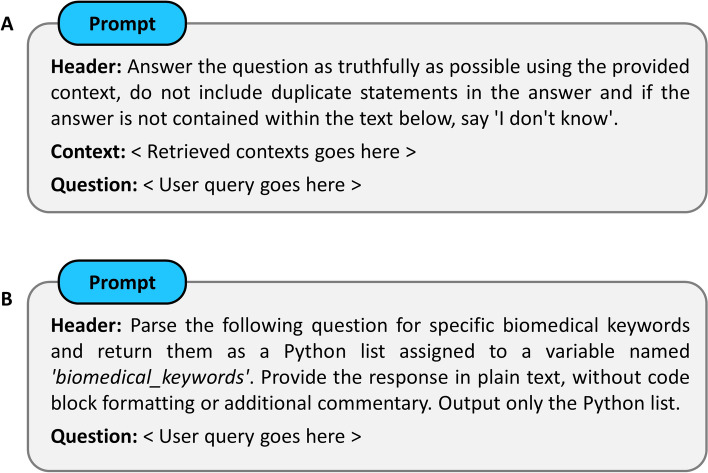
Architecture of our prompt—Custom instructions in the header guides an LLM to produce desired outcomes. **A** Reinforces an LLM to produce improved responses by combining the elements of information retrieval and generative AI. **B** Exploits LLM’s zero-shot reasoning capability to transform it into an automatic keyword extractor module

In vector DB, every vector points to a corresponding chunk. Thus, by identifying *top k* similar vectors, we can extract these chunks to construct the user prompt. In this prompt engineering approach, we first extract the *top k* relevant chunks, then wrap them around the user query. The LLM is subsequently instructed to find the answer within these chunks only, and if the answer is not found then respond with ‘I don’t know’ (see Fig. 4A).

However, it is important to note that this choice of *top k* is very much dependent on the context lengths provided by LLMs (Table 1). Therefore, ideally the top chunks should contain relevant information to provide a satisfactory answer to the question. Yet, this is not always the case (See Results, section “PES1 struggles with optimal prompt generation in relatively large knowledge bases”), which makes the QA bot perform poorly. Thus, we developed a second prompt enhancement approach, as described in the next section.

**Table 1:** LLMs and their context lengths

Model	Context lengths	Max fit
GPT-3	4 K tokens ≈ 16 K chars	8 chunks (7 context + 1 answer*)
GPT-4	8 K tokens ≈ 32 K chars	16 chunks (15 context + 1 answer*)

*Assuming generated answer length is $\le$ 2000 characters

### Context retrieval based on keyword frequency ranking (PES2)

In order to find the most relevant chunks for a given query, we implemented a strategy that extracts keywords from the user’s query and ranks paragraphs/chunks higher if they contain a higher keyword count. The keyword extraction can be done either manually or automatically. Using well-designed and explicit instruction headers within the prompt, it is possible to make an LLM function as automatic *keyword extractor* (see Fig. 4B). Our QA bot implementation offers both options. For automated extraction, the LLM outputs the keywords as a python list. However, in some scenarios, it might be necessary to exclude certain keywords or combine adjacent keywords into a single item. In such cases a manual approach is more appropriate. For manual extraction, queries are prefixed with a ‘#’ symbol for ease of programming and keywords are marked using double asterisks. For example, **#**Find all results that connect **NuA4** with **meiosis**.

**Fig. 5:**
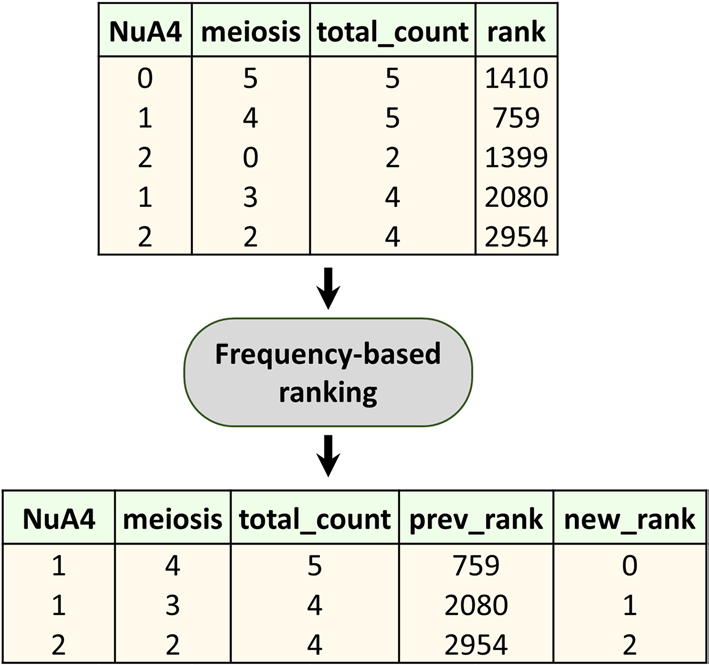
Re-ranking chunks—‘NuA4’ and ‘meiosis’ are keywords extracted from a query*: Find all results that connect NuA4 with meiosis*. Each row represents a paragraph or chunk from research papers, with columns displaying both individual and total frequencies of the keywords. The upper table shows chunks that are initially ranked according to cosine similarity computed between the query and each chunk in the vector DB. The bottom table depicts the re-ranking of the same chunks based on their keyword frequencies. The ranking starts at 0, indicating that the topmost relevant chunk is ranked at the 0th position

**Fig. 6:**
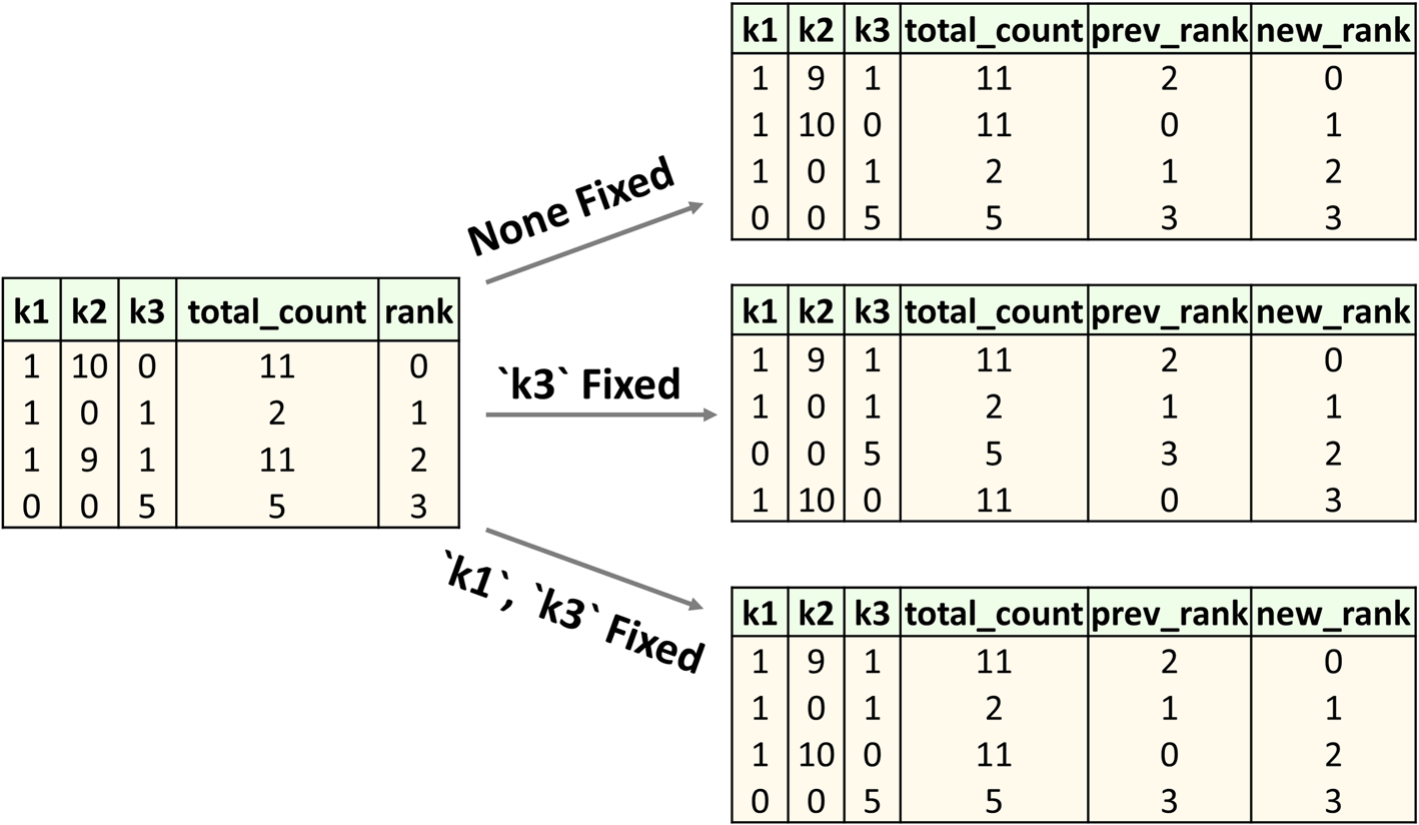
Variants of ranking criteria—Each row represents a text chunk, columns indicate both individual and total frequencies of three keywords: k1, k2 and k3. The left table presents chunks initially ranked according to cosine similarity. The successive tables on the right depict the re-ranking of these chunks under different ranking criteria: without any fixed keywords (None Fixed), with one keyword (‘k3’) is fixed, and with multiple keywords (‘k1’, ‘k3’) fixed, respectively

The approach is based on the consideration that chunks that have a higher frequency of the extracted keywords are more likely to contain useful contextual information to generate a high-quality response. For example, suppose the upper table in Fig. 5 was obtained after ordering chunks using cosine similarity-based ranking. According to our considerations, the second row should clearly have come first. To correct that, we re-ranked the chunks by elevating the most relevant chunks based on keyword frequencies, ensuring key information surfaces to the top (Fig. 5, lower table). To illustrate our approach further, we present additional examples of ranking variants in Fig. 6. In one mode, we only keep chunks in which all keywords appear at least once and in the other we rank chunks based on the cumulative frequency of all keywords. In addition, other kinds of ranking are also possible, as described below.

- None fixed: A chunk is ranked higher if it contains all the three keywords over a chunk that does not. Even if the total frequency count is the same or even higher. When two chunks have nonzero values for all keywords, the one with higher ‘total_count’ is ranked higher. In this mode equal importance is given to all the keywords.
- One fixed: More importance is given to chunks that have nonzero values in the fixed keyword column which in this case is ‘k3’. This way of ranking chunks is based on Boolean expressions (k1 OR k2 AND k3), similar to searches in PubMed.
- Multiple-fixed: This mode prioritizes chunks containing nonzero values for several specified keywords. Extending the one-fixed approach, it allows for finer chunk evaluations by focusing on multiple key terms. Thus, this method ranks chunks higher when they contain all designated fixed keywords, thereby enhancing specificity in data sorting.

Once the chunks have been extracted and a context for answering the user query is created, processing takes place via LLM.

### Processing queries by accessing LLMs via API

The extracted context is wrapped around the user query and a prompt for LLM is created. We use the API services from OpenAI for customized interaction. In the prompt header, the LLM is explicitly instructed to look for the answer only within the given context and if the answer cannot be found then it should return appropriate response, such as ‘I don’t know’ (see Fig. 4A). Thus, by instructing the model to search within the provided passages, we give it access to both the latest and most relevant data, while also leveraging the LLM’s contextual understanding and ability to generate human-like responses.

Our pipeline (Fig. 1) can be divided into two main modules: one for designing relevant prompts and the other for generating meaningful responses. At the heart of the *prompt enhancement* module lies the text embedding, which aims to capture the essence of text in a compressed representation that can be utilized to obtain relevant contexts. At the same time, the core to the *answer generation* module is the LLM responsible for generation human like responses. As users of AI models, we are bound by certain constraints: For instance, creating a proprietary LLM is currently prohibitively expensive for most users. Despite that, we can still influence the outcome of user queries by tweaking the other aspects of our pipeline. Our hypothesis is that text embedding has a significant impact on the final response generated by LLMs. This is because we determine the context for our prompts through these embeddings and their similarities with the query.

### Experiments: influence of text embedding on answer quality

Before discussing the experiment, it is important to briefly note some of the key aspects of text embedding. Vector embeddings can be seen as a form of compression, a way of capturing the essence of text, i.e., semantic similarity between words or sentences in a format that a machine can understand and manipulate. It has been shown that the dimensionality of embeddings can impact model's understanding of semantic relationships [24, 32]. There are several text Embedder models available, and they embed the texts into different dimensions. For example, the OpenAI model embeds text into 1536 dimensions. A larger number of dimensions might capture more information, but it also requires more computational resources to process and store. Higher-dimensional vectors might also include dimensions that capture noise rather than useful information, leading to overfitting. Conversely, reducing the dimensionality might lead to information loss, but it can help focusing on the most important aspects of the text’s properties and result in models that are more efficient and less prone to overfitting. Consequently, there is a trade-off which a practitioner needs to optimize. Also, text embeddings are domain sensitive because the meaning of words can differ based on context. For example, “cell” in a biology text refers to a basic unit of life, but in a telecom context, it refers to a cellular network. Therefore, researchers have published several embedding models that are fine-tuned/re-trained on data from a specific domain, such as BioBERT which is BERT model pre-trained on large-scale biomedical texts. BioGPT is a GPT model in its core but again trained on vast amounts of texts from biomedical literature. The latest addition to the biomedical domain specific model is MedCPT, which is a hybrid model that includes both a retriever and a re-ranker component that are pre-trained on large number of query-article pairs from PubMed search logs.

**Fig. 7:**
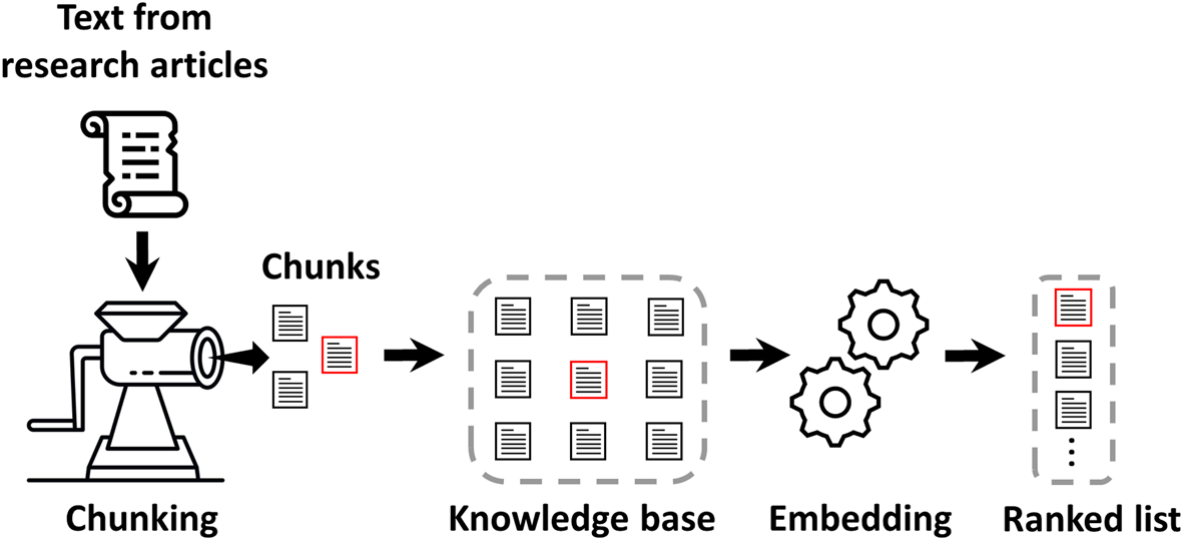
Experiment to determine optimal embedding model—Text from research papers is broken into smaller chunks to construct a knowledge base. A key chunk (which contains the answer) is highlighted within a red rectangle. The objective is to evaluate which embedding model assigns the highest rank to the key chunk

Based on these facts, we hypothesized that different text embedder models can influence the quality of answers generated by LLMs. We therefore assessed which of the four models: BioBERT, BioGPT, MedCPT and text embedder ADA from OpenAI works best for our knowledge management use case. The experiments were performed as follows: we first created a knowledge base in which publications from a specific topic are stored after recursively splitting them into small chunks of 2000 characters (chars; as depicted by the small boxes in Fig. 7). This specific chunk size was determined arbitrarily. Experimenting with various chunk sizes may be helpful to evaluate how they influence the quality of the generated answers. We then formed a question, already knowing which chunks contain the answer, which we refer to as *key chunks*. Next, we modified our knowledge base to contain only one key chunk (while the rest were non-key chunks) and we asked which embedding model can yield vectors corresponding to the key chunk and our query, so that the similarity between them is the highest. In other words, we evaluated which embedding model can rank the key chunk the highest, keeping in mind LLMs like GPT-3 and GPT-4 can only consider the top 8 and 16 chunks, respectively (see Table 1). This entails that any chunk ranked lower than these thresholds will be missed by the corresponding LLMs.

## Results

### PES1 struggles with optimal prompt generation in relatively large knowledge bases

In our first setup, the knowledge base contained just 7 publications and one known key chunk from one paper which we refer as the *key paper*. Our query was: *Find all results that connect NuA4 with meiosis*. We found that both BioBERT and BioGPT ranked the key chunk 2nd and MedCPT ranked it 1st. OpenAI's ADA, though, ranked it 9th. So, if we use ADA with GPT-3's limit, then the key chunk would be missed and hence the answer.

Next, we further evaluated whether this pattern persisted with larger namespaces, using the same query as above. Hence, in our second experiment, we created a larger knowledge base comprising approximately 200 papers, including the key paper from the first experiment. For the same query, we compared the retriever performance of different embedder models based on the top ten retrieved chunks. Again, MedCPT outperformed the others by placing the key chunk at the 3rd position while no other embedder model was able to retain the key chunk within their top ten retrieved chunks. The closest one was BioBERT that placed the key chunk at the 29th position which was beyond the reach of both GPT-3 and GPT-4's context limits. Furthermore, we inspected the chunks ranking above the 29th place and found that they lacked sufficient context to answer the question as effectively as the key chunk. This demonstrate that the choice of embedding has a significant impact on the answer. Thus, to further assess the retrieval power of PES1, we selected MedCPT, the best performing embedder model, and evaluated it on questions covering a diverse range of concepts within chromatin and computational biology (See Table 2). Some of the questions are listed in Table 3. In this test, PES1 did not perform well, as for most of the questions it gave “I don’t know” (Wrong) answers and, for some of the questions the answers were either not complete, lacking depth and comprehensibility. Upon inspecting the top ten retrieved chunks for each question, we found that the key chunks containing the answer was missing for most of the questions. These observations highlighted the need for boosting the key chunk's visibility. Therefore, to address this issue, we developed PES2.

**Table 2:**
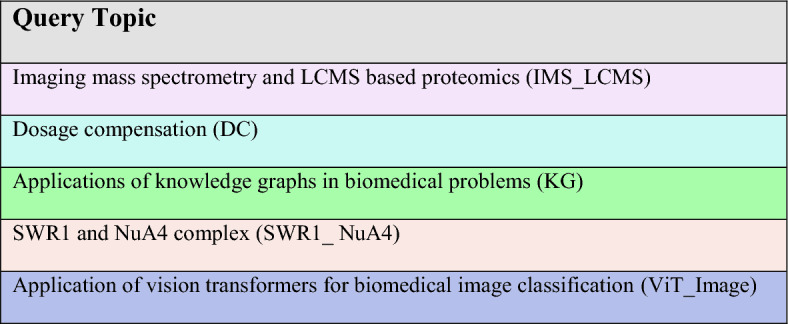
Queries from various biomedical topics used to evaluate PES1 and PES2

Query topics are color-coded for clearer categorization and abbreviations are given to efficiently refer them in the text.

**Table 3:**
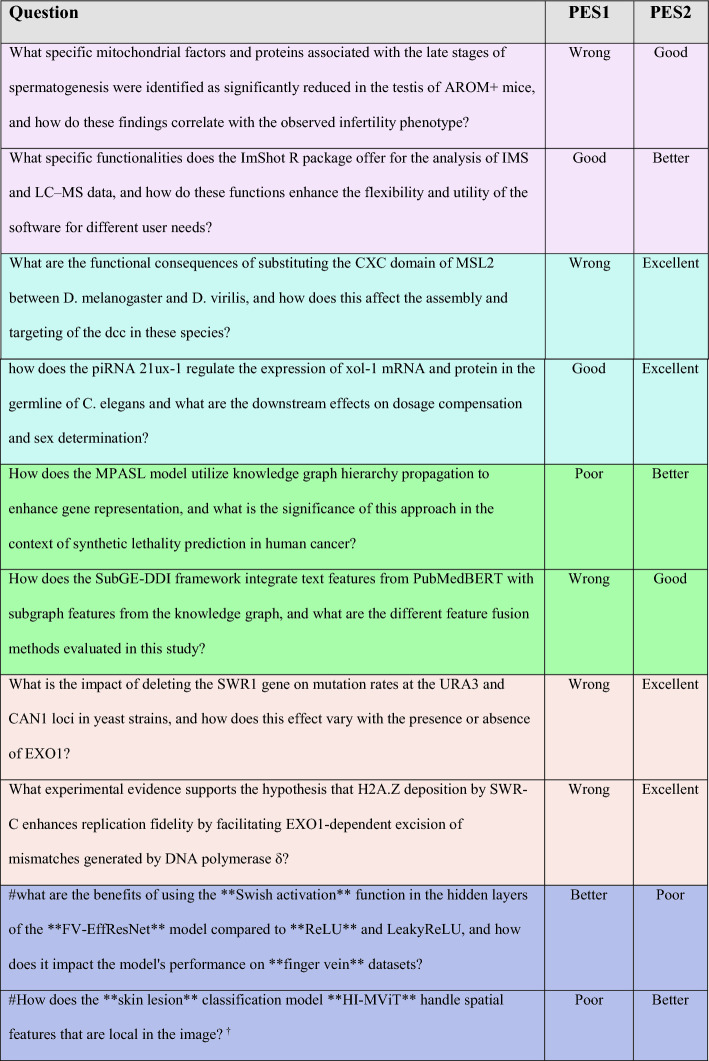
Comparison of LLM-generated responses based on PES1 or PES2 for questions from topics described in Table 2.

Due to space constraints, only 10 questions are shown while the answer, citation columns are omitted. The complete responses generated by LLM for all 50 questions are available in the supplemental file (See Additional file 1). Queries are color-coded to indicate their connection with diverse topics, as illustrated in Table 2. ^†^Manually marking keywords (See the above section titled "Context Retrieval based on Keyword Frequency Ranking (PES2)" under Methods) aided in obtaining a better response for this query.

### PES2 improves prompts by boosting the visibility of relevant chunks

**Fig. 8:**
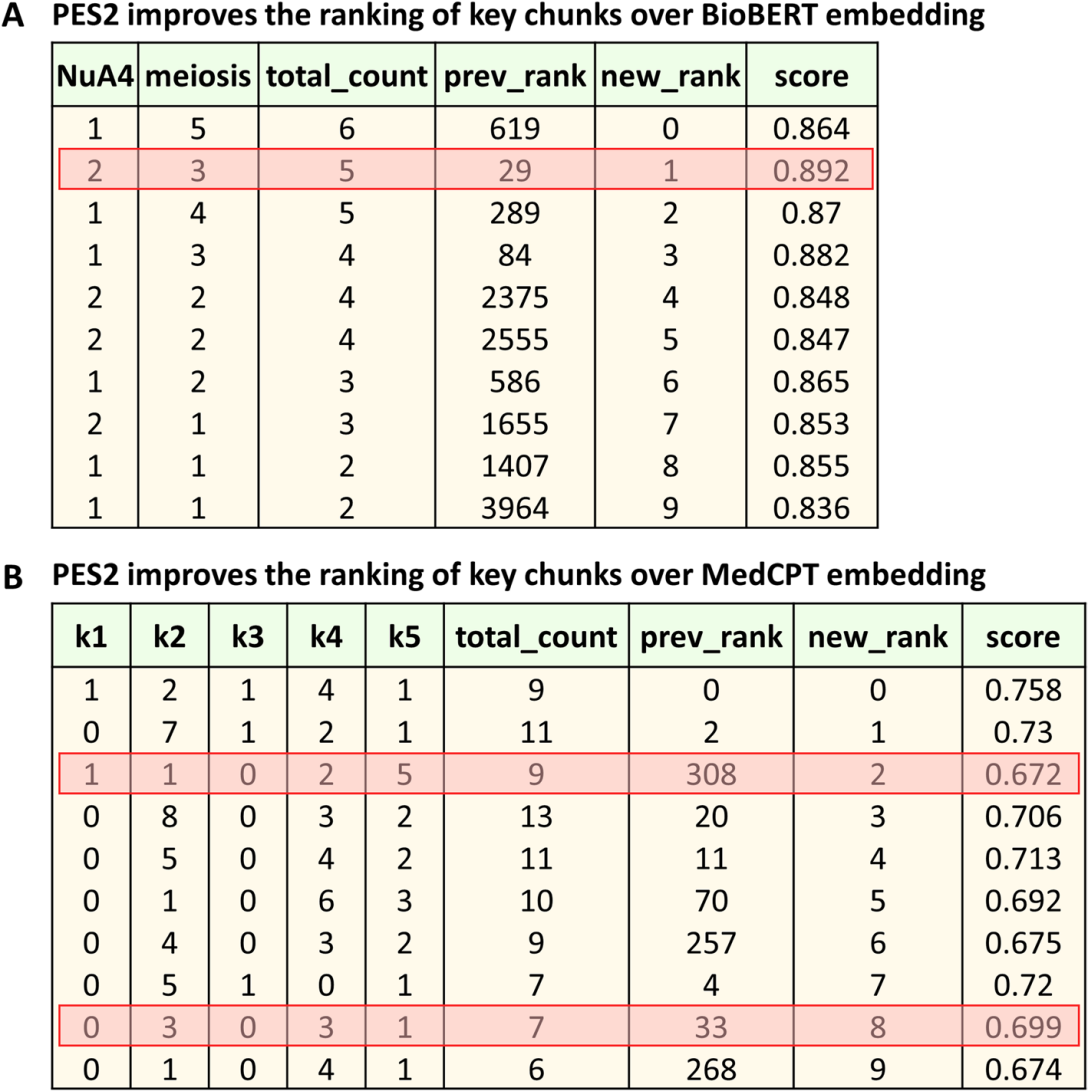
Ranking chunks by keyword frequencies (using PES2) ranks key chunk(s) higher—**A** Top ten chunks retrieved using the BioBERT model for the query *Find all results that connect NuA4 with meiosis*, with a ranking of zero indicating the highest position. The first two columns show the frequencies of extracted keywords: *NuA4* and *meiosis* in each chunk. The key chunk (highlighted using red rectangle), originally ranked 29th using cosine similarity scores, is elevated to 1st after applying the keyword frequency-based ranking method. The knowledge base size is moderate, with approximately 200 papers. **B** Top ten chunks retrieved using the MedCPT model for the query *What specific mitochondrial factors and proteins associated with the late stages of spermatogenesis were identified as significantly reduced in the testis of AROM* + *mice, and how do these findings correlate with the observed infertility phenotype?* with a ranking of zero indicating the highest position. The first 5 columns show the frequencies of extracted keywords: *mitochondrial factors(k1)*, *proteins(k2)*, *late stages of spermatogenesis(k3)*, *testis(k4)*, *and AROM* + *mice(k5)* in each chunk. The key chunks (highlighted using red rectangles), originally ranked 308th and 33rd using cosine similarity scores, are elevated to 2nd and 8th positions, respectively, after applying the keyword frequency-based ranking method. The knowledge base size is large, with approximately 1000 papers

We applied PES2 to our previous knowledge base comprising approximately 200 papers, using the same query as before. As shown in Fig. 8A, the re-ranking process significantly improved the position of the key chunk, lifting it from the 29th to the 1st place (ranking of zero indicating the highest position). This performance surpassed the initial ranking given by BioBERT and matched the retrieval performance of top-performing embedding algorithm MedCPT when using PES1, for this moderately-sized knowledge base. Next, we applied PES2 to answer a more challenging and specific question: *What specific mitochondrial factors and proteins associated with the late stages of spermatogenesis were identified as significantly reduced in the testis of AROM* + *mice, and how do these findings correlate with the observed infertility phenotype?* This time, we used a much larger knowledge base comprising approximately 1000 papers. We compared the retrieval performance with MedCPT, as it had performed reasonably well in the moderately-sized knowledge base. Interestingly, MedCPT failed to retrieve the key chunks into its top ten retrieved chunks, in this larger knowledge base. Applying PES2 improved the ranking of the key chunks, elevating them from 308th and 33rd to 2nd and 8th positions (counting 0 as first), respectively, as depicted in Fig. 8B. Additionally, PES2 also ranked up other relevant chunks which were low-ranking before.

### Performance evaluation

To further evaluate PES1 and PES2, we assessed the quality of answers generated by the LLM for 50 questions (see Additional file 1). These questions were selected from the diverse range of topics presented in Table 2. For this evaluation, we designed an experiment simulating a “needle-in-the-haystack” scenario, as follows: For questions related to a specific topic, we created a knowledge base or namespace containing a large number of papers, where papers on the specific topic were in the minority, and the majority were related but from different topic(s). For example, for questions related to *IMS_LCMS*, we created a large namespace with approximately 1000 papers. Of these, only 6 papers were specific to it while the rest covered related but different topics, such as dosage compensation. For robust evaluation, we created separate namespaces comprising a large number of papers for each query topic mentioned in Table 2. The article distribution among the query topic (QT) and other topics (OT) in these namespaces is summarized in Table 4. For this set of 50 questions, we used GPT-4o, which is the most advanced model from OpenAI. We then asked specific questions derived from papers belonging to different query topics, we refer those papers as *key papers*. These papers were minorities in every namespace (See Table 4), thereby simulating a “needle-in-a-haystack” setup.

**Table 4:** Article distribution within namespaces

Namespace	QT	OT	TP	PQT	KP (%)
1	IMS_LCMS	Dosage compensation	946	6	0.63
2	DC	Chromatin proteomics	1437	5	0.35
3	KG	Dosage compensation	945	5	0.53
4	SWR1_NuA4	Dosage compensation	945	5	0.53
5	ViT_Image	SWR1 and NuA4	423	8	1.89

QT → Query Topic (Acronyms are defined in Table 2); OT → Other Topic

TP → Total papers; PQT → Papers from Query Topic

KP (%) → Percentage of key papers in a namespace

We then employed PES1 and PES2 to seek answers to those questions, evaluating their retrieval performance using the following criteria: For every query, we computed the fraction of the top ten retrieved chunks that originated from the key papers, for each retriever (PES1 and PES2). In other words, we estimated the retrieval precision of different retrievers for each question, as given in the following equation:

2 $$Precision@10= \frac{Number \;of\; relevant\; chunks\; in\; top\; 10\; retrieved\; chunks}{10}$$

**Fig. 9:**
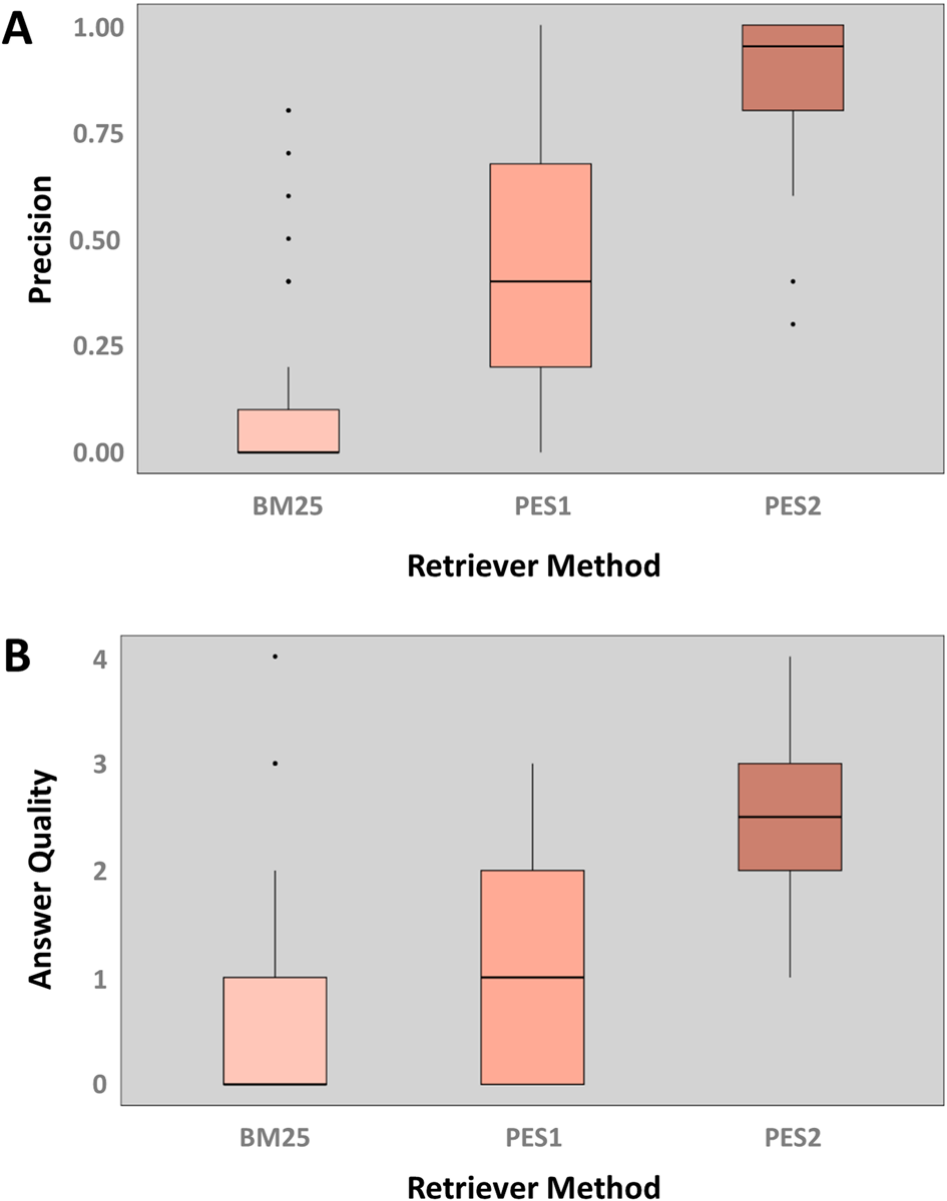
Evaluating performances of BM25, PES1, and PES2—**A** Based on Precision@10: For each query, we computed (using Eq. 2) the fraction of the top ten retrieved chunks that originated from the key paper for each retriever. PES2 demonstrated the highest precision among the three retrievers, with a median of 0.95. **B** Based on LLM-generated answer qualities: We determined the quality of LLM-generated answers through manual assessment. For the quantitative evaluation, we categorized the generated answers into five categories: Excellent (4), Better (3), Good (2), Poor (1), and Wrong (0). Using PES2 led to the highest quality answers among the three retrievers, with a median score of 2.5

For a more robust evaluation, we also compared PES1 and PES2 with BM25 which is a commonly used baseline model in information retrieval and NLP. It is a ranking function that evaluates document relevance to a query by combining term frequency, inverse document frequency, and document length normalization [33, 34]. Due to its simplicity and efficiency, it is a robust and reliable method for comparing more complex or novel approaches. The retriever precision results are plotted in Fig. 9A, clearly showing that PES2 is the best and BM25 is the worst performing retriever for these 50 questions.

Furthermore, we manually evaluated the quality of LLM-generated answers for every question from each retriever using the following criteria: relevancy, clarity/comprehensibility, and depth of information/completeness. We then categorized the generated answers into five categories: Wrong (0), Poor (1), Good (2), Better (3), and Excellent (4). The corresponding category scores are given in between parenthesis with “Excellent” answers receiving the highest score of 4 and the “Wrong” answers receiving the lowest score of 0. The categories were defined as follows:

- Wrong: “I don't know” answers.
- Poor: Incomplete answers lacking depth, informativeness, specificity, or clarity.
- Good: Clear and comprehensible answers.
- Better: More informative, comprehensible, and specific answers.
- Excellent: Comprehensive and highly informative answers.

The results that are plotted in Fig. 9B, using the scores for each question, for each retriever, indicate that PES2 produced high quality answers for most of the 50 questions, outperforming the other methods.

During the evaluation, the input prompt consisted of the following three pieces of information (see Fig. 4A):

- Header: This contains instructions that an LLM is supposed to follow.
- Context: This is where the context to answer a question is placed.
- Question: This is a placeholder for user questions.

As shown in Fig. 4A, we explicitly instructed the LLM in the header not to make up an answer and seek it only within the provided context and if, and only if, an answer is not contained in the context then respond with ‘I don’t know’. It is clear from Table 3 and the supplemental Table (see Additional file 1) that PES1 struggled to generate meaningful prompts as it responded with ‘I don’t know’ or generated poor answers for most of the questions. Furthermore, the information content in contexts retrieved by PES1 was not relevant enough to produce good quality answers for several questions. This was even worse for questions that seek extremely specific details. On the other hand, with PES2, we obtained good quality answers to the majority of the questions. We provided all the detailed answers along with their references in the supplementary table (Additional file 1). Furthermore, the parameter settings used to evaluate both PES1 and PES2 in the WeiseEule app are provided in Additional file 2, which also includes step-by-step instructions for setting up WeiseEule as a localhost application on user machine.

### Salient features of the WeiseEule app

**Fig. 10:**
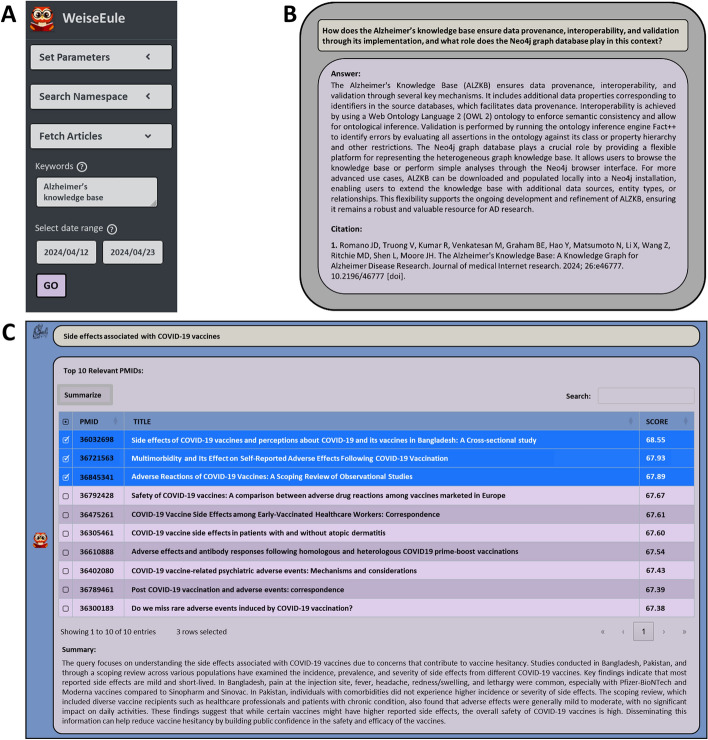
Salient features of the WeiseEule app—**A** Users can download article texts from PubMed by entering keywords in the provided search box. This feature enables users to create namespaces/knowledge bases with the articles published during specified date ranges. **B** Users can query a selected namespace with relevant questions. Answers are supported by original references, and the contexts used to produce the answer are also provided. **C** Users can find relevant PubMed articles corresponding to their keywords/queries. This panel shows the top ten relevant PMIDs matching the query: *Side effects associated with COVID-19 vaccines*. PMIDs are sorted in descending order based on their relevance scores computed through a combination of vector similarities and deeper semantic comparisons
  One advantage of the date range feature is that users can update their namespaces periodically or whenever they want. To update the existing namespace with latest research papers, users need to first select a namespace on the app GUI and then input the keywords and a date range, and hit the *GO* button. Currently, the namespace update is done manually, however, in the future, we will make this feature automatic. In addition, users can also use their PDFs to create namespaces. The namespace creation from user uploaded PDFs is currently done using a Python script which comes with the WeiseEule App repository. Step by step instructions on how to use the script is given in Additional file 2. In the next version of the app, we will also support this feature directly via app GUI.
- Answer questions with source and context: In the chat panel (See Fig. 10B), users can query a namespace after selecting an LLM and the relevant namespace from the dropdown menu in the sidebar (See supplementary Fig. S3) in the sidebar. Answers are backed by references and the contexts used to generate them (See Fig. 10B). The app also allows users to experiment with different PES methods, LLMs and chat completion settings (See supplementary figures S3, S4).
- Find relevant articles: This feature helps users to find articles relevant to a set of keywords or queries in the *Search* panel. The app presents the search results as an interactive table with three columns: the first column lists the PubMed IDs (PMIDs) relevant to the query/keyword(s), the second column shows the corresponding article titles, and the third column presents the relevance score, which indicates how closely the abstracts of the retrieved PMIDs match the query/keyword(s). This score is determined through a combination of vector similarities and deeper semantic comparisons [29], and it is used to sort the retrieved PMIDs in descending order (See Fig. 10C).
  We anticipate this feature to be useful in multiple use cases. For instance, if a user is writing a review on COVID-19 and wants to discuss about the vaccines and their side effects, in a section of the manuscript. By using the WeiseEule app, the user can retrieve PMIDs, and the corresponding article titles ranked according to their relevance to the query (as exemplified in Fig. 10C). Thus, this allows the user to focus on the most relevant publications, a feature that is particularly valuable for reviewing topics with extensive literature. In addition, the app provides a summary of the selected abstracts, and the PMIDs are hyperlinked to their respective pages on PubMed. Hence this also allows the user to verify the results, as well as download the citation files easily.
  This feature can also help users to identify publications that can be cited to support text they have already written. To this end, the user formulates a search query by entering text parts they want to support with a citation in the *Search* panel. The app will then return a list of articles most relevant to the search keywords/query, from which the user can select suitable articles to cite. Note that since the ‘*Find relevant articles*’ feature only provides access to the relevant abstracts, it is more suitable for assistance during review or grant writing or to find articles to cite. On the other hand, the ‘*Answer questions with source and context*’ feature described earlier is more suitable for seeking very specific information buried inside the full text of articles.
  To implement this feature, we used the embeddings of PubMed articles from NCBI, generated using the MedCPT article encoder. On their FTP server,^2^ NCBI organized the embedding files into batches, each containing about 1 million articles. For instance, batch 0 includes PMIDs ranging from 0 to 999999. However, each batch may not contain exactly 1 million articles due to some articles being missing. Apart from embeddings files for each batch, the FTP link also provides two additional files necessary to implement this feature. One file is for the 1 million PMIDs corresponding to the embedding vectors, and the other one is a python dictionary mapping these PMIDs to their abstracts. These files are large, with each batch averaging approximately 4 GB. Currently, there are 38 such batches of data corresponding to approximately 38 million PubMed articles. Searching this entire dataset is challenging, so we restricted the search feature to one batch (1 million articles), at a time. Users need to download the files and keep them in the *MedCPT_Embeddings* folder, inside the app folder, and mention the batch number when using the search feature. For example, if the search query is “Side effects associated with COVID-19 vaccines” and users wants to search batch 36, they should enter “Side effects associated with COVID-19 vaccines #36” in the panel.
  However, we acknowledge that batch-wise searching is time consuming and sub-optimal and for some queries may not yield the best hits. Therefore, we are working on Elasticsearch implementation for this feature to be incorporated in the next WeiseEule release. Elasticsearch is a powerful search and analytics engine designed for efficient handling of large volumes of data [35]. It is particularly useful for applications involving complex search queries and full-text search across massive datasets, which closely matches our use case.
- Flexible software design: A key feature of WeiseEule app is the flexible nature of its software design, consisting of two main components: Frontend and Backend. Frontend manages the user interface (UI) for different features, while the backend implements the logic to actualize these features. The two ends communicate via WebSocket protocol which provides full duplex communication, meaning either end can send messages at any time (See Fig. 11). This setup enables the LLM to stream responses token by token from the backend to the frontend, enhancing the user experience significantly. Moreover, the code for UI and the corresponding API endpoints are completely segregated and modular, making it fast and easy to add or remove UI features or API endpoints.

The WeiseEule app was designed to facilitate efficient information retrieval from biomedical articles. Here are some of its key features:

- Namespace creation: This feature allows users to fetch PubMed articles on the topic(s) of interest and create a namespace in vector DB that serves as a queryable knowledge base. The app provides a textbox for inputting keywords. For example, if a user is interested creating namespace for *Alzheimer’s knowledge base*, then this can be typed in the provided input box, followed by selecting a date range (See Fig. 10A), and finally hit the *GO* button. Then the app will download papers in which the keywords appear either in the titles or in the abstracts, provided they were published within the given date range.

**Fig. 11:**
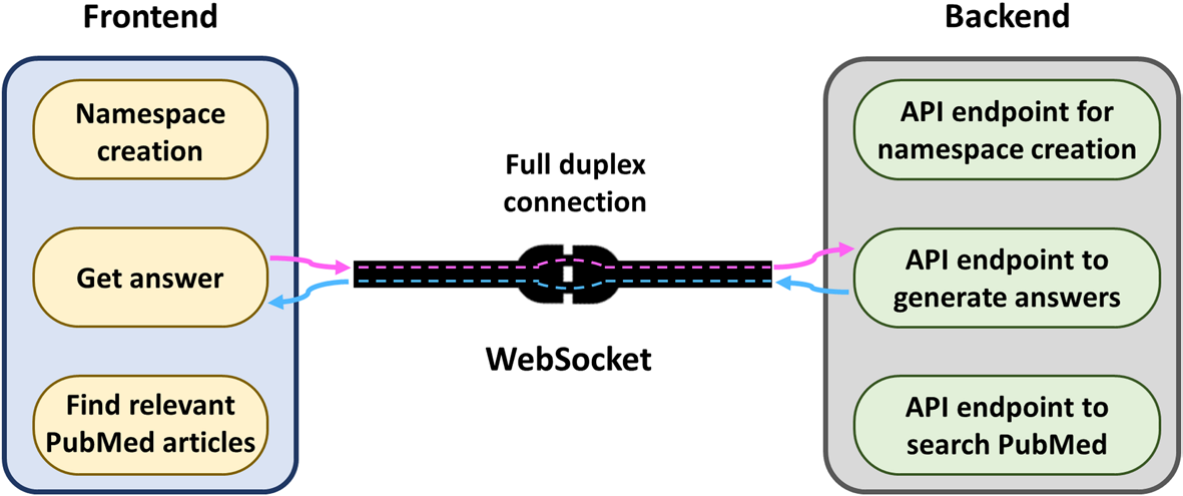
Flexible design of the WeiseEule app—The WeiseEule app features a flexible software design with two main components: the frontend, which handles the user interface (UI), and the backend, which implements the logic for various features. These components communicate via WebSocket protocol, enabling full-duplex communication for real-time data exchange. This architecture allows the LLM to stream responses efficiently from the backend to the frontend, significantly enhancing the user experience. The modular and segregated code structure of the UI and API endpoints facilitates quick and easy addition or removal of features

## Discussion

### Performance of PES1

Our experiment with different embedding models in PES1 highlights an interesting aspect of text embedding. The performance of BioBERT, BioGPT, MedCPT, and OpenAI's ADA in ranking key chunks from biomedical literature can be influenced by a number of factors, including their design, training data, and algorithms.

#### BioBERT's performance

BioBERT was pretrained on a large corpus of biomedical texts, which likely gives it an edge in understanding and contextualizing biomedical queries. However, it ranked the key chunk much lower (29th place) in a bigger knowledge base than it did in a smaller setting (2nd place). This suggests a limitation in its retrieval capabilities as the complexity of knowledge base increases. This indicates that, as the size of the knowledge base grows, even a domain-specific model like BioBERT might struggle with the increased diversity and complexity of the data, leading to lower quality information retrieval.

#### BioGPT's performance

BioGPT, similar to BioBERT, is tailored for biomedical content but there is an important distinction: the focus of the BERT model is to understand the meaning of words in relation to the entire text, whereas GPT excels at text generation and completion. So, as a generative model, BioGPT approaches embedding of texts differently which in turn influences ranking.

#### MedCPT's performance

MedCPT is designed for zero-shot biomedical information retrieval and excels at capturing complex semantic relationships within texts. However, in some “needle-in-the-haystack” situations, where the objective is to find specific information within a large corpus, it might underperform. Most of the questions in our evaluation set were specific, for example,

- *What specific mitochondrial factors and proteins associated with the late stages of spermatogenesis were identified as significantly reduced in the testis of AROM* + *mice, and how do these findings correlate with the observed infertility phenotype?*

Or

- *What experimental evidence supports the hypothesis that H2A.Z deposition by SWR-C enhances replication fidelity by facilitating EXO1-dependent excision of mismatches generated by DNA polymerase δ?*

The “I don’t know” or “Poor” responses from PES1 (using MedCPT embedding model) to many such questions indicate that, in scenarios requiring highly specific information needs, MedCPT embeddings may contain semantically related but contextually less relevant information, which would reduce precision.

#### OpenAI's ADA performance

Being the worst performer, OpenAI's ADA highlights a broader issue with using general-purpose embeddings in domain-specific tasks [36–38]. ADA is trained on a variety of texts, not just on biomedical literature. The comparison of ADA with targeted models like BioBERT or MedCPT highlights that, generalist training approaches appear to limit the model’s ability to understand and prioritize the complexities of biomedical text, resulting in poorer performance in specialized tasks. ADA’s ranking of the key chunk beyond the reach (9th place) of GPT-3 even for such small set of chunks emphasizes the challenge that generic embeddings face in highly specialized domains. Fine tuning ADA on a large biomedical corpus may make it more sensitive to the specific cues that indicate relevance in biomedical texts but doing that comes with additional expenses in terms of expertise, time, and money.

### Performance of BM25

To rank documents, BM25 uses term frequency-inverse document frequency (TF-IDF), and document length normalization. Although this method works well for numerous general information retrieval tasks, it might not be appropriate for highly specific queries. We speculate that BM25 may have had difficulties in our “needle-in-the-haystack” scenario because the number of related but less relevant papers far outweighed the number of important papers. Thus, it may be difficult to achieve the necessary nuanced relevance using the straightforward term frequency model.

### Performance of PES2

By re-ranking text chunks based on the frequency of specific keywords extracted from user queries, PES2 prioritizes content that is apparently more relevant to the users' informational needs.

**Strengths of the keyword frequency-based re-ranking**

- Improved relevance: The results show that our re-ranking strategy significantly improves the relevance of retrieved chunks compared to the embedding-based approaches which mainly rely on cosine similarity measures in vector space. PES2 more efficiently surfaces information that users are likely to find helpful by directly aligning the retrieval process with the explicit keywords in user queries.
- Simplicity and efficiency: In contrast to intricate NLP methods that necessitate substantial computational power and advanced AI models, keyword frequency analysis is reasonably easy to apply and computationally effective. This approach is therefore particularly appropriate for applications where quick response times are essential or resources are scarce.
- Flexibility: The approach offers considerable flexibility, allowing for easy adjustment of the re-ranking algorithm to accommodate different keyword selection schemes or to incorporate more relevance signals as needed.

The PES2 approach represents a promising advancement toward the creation of more relevant and responsive biomedical question-answering systems. This method provides a practical trade-off between retrieval effectiveness and computational efficiency by emphasizing the explicit signals supplied by user queries. However, realizing its full potential will require addressing its current limitations:

- Handling of synonyms and related terms: A drawback of the PES2 approach is its dependence on precise keyword matches, which might leave out domain specific nuance like synonyms and related terms. In biomedical texts one keyword may have multiple synonyms, for example, Heterochromatin protein 1, Hp1a and Su(var)205 all refer to the same entity.
- Typos and Spelling Variations: Spelling errors can have a substantial impact on the retrieval process and may result in the exclusion of valuable information.
- Contextual relevance: Although keyword frequency can be used as a stand-in for relevance, it does not take the context of a keyword's appearance into consideration. As a result, this approach may give preference to sections that have a high keyword density but provide little insight into the user's actual query.

To address these limitations, future work could investigate the integration of semantic analysis techniques to better understand the context and meaning behind user queries. We could also explore integrating query expansion techniques which, in association with resources such as the Unified Medical Language System (UMLS) could help broadening the search query for biomedical texts by including synonyms or related terms. This could increase the likelihood of finding relevant chunks, even in cases where the exact keywords are misspelled. Typos and slight variations in the user's query can be explored by fuzzy matching algorithms. To locate close matches to the keywords in a database, methods such as Levenshtein distance (a type of edit distance) can be employed. This way, relevant chunks can still be retrieved based on the closest matches to the intended keywords, even if misspelled. To improve contextual relevance, future work could include the integration of knowledge graphs. The graph can be used to enable semantic search functions. Understanding the semantic relationships between entities can be done by looking at the graph's structure rather than just depending on keyword frequencies. This can help in ranking chunks not just by the occurrence of keywords but by the relevance of the context in which those keywords appear.

## Conclusions

Our findings emphasizes the importance of integrating external knowledge with generative AI to improve biomedical QA tasks. Through our proof-of-principle study, we demonstrated the significance of prompt enhancement strategy based on explicit signals in user’s query over traditional text embedding based context retrieval approaches. This method not only improved retrieval precision, with a median Precision@10 of 0.95, but also significantly enhanced the quality of LLM-generated answers, achieving a median quality score of 2.5.

We also highlighted the importance of namespaces in streamlining the search, enabling users to experiment with different chunk sizes for enhanced precision in answers. This feature in the WeiseEule app enables users to create custom knowledge bases by fetching full texts from PubMed and user-uploaded PDFs, providing complete control over the information that goes into the LLM. Additionally, the app includes an article recommendation feature to assist users during review or grant writing, and in finding articles that can be cited to support already written text.

Lastly, our method presents a viable strategy to strike a balance between retrieval efficiency and computational demands. Moving forward, unlocking the full potential of this method will depend on addressing its current constraints. However, the insights derived from this investigation hold promise for broadening the horizons of NLP research and its application in solving complex QA challenges, especially in specialized fields like biology and biomedicine.

### Supplementary Information

Additional file 1. An Excel file comparing BM25, PES1, and PES2 based on LLM-generated answers for fifty questions. The file contains five sheets, each corresponding to the query topics mentioned in Table 2. The sheets are named using the abbreviations introduced in Table 2.

Additional file 2. Step-by-step instructions for setting up WeiseEule as a localhost application on user machine.

## Data Availability

The data used to construct namespaces for the performance evaluation of PES1 and PES2 are available at: https://github.com/wasimaftab/WeiseEule-LocalHost/tree/main/Data_Namespace. The WeiseEule application, implemented as a localhost web application, can be accessed at the following GitHub repository: https://github.com/wasimaftab/WeiseEule-LocalHost.

## Abbreviations

NLP: Natural language processing
LLMs: Large language models
QA: Question-answering
DB: Database
APIs: Application programming interfaces
PESs: Prompt enhancement strategies
RAG: Retrieval-augmented generation
KB: Knowledge base
BERT: Bidirectional encoder representations from transformers
GPT: Generative pre-trained transformer
PES1: Context retrieval based on vector similarity ranking
PES2: Context retrieval based on keyword frequency ranking
UMLS: Unified Medical Language System
MedCPT: Medical contrastive pre-trained transformer

## Footnotes

1 WeiseEule: /ˈwaɪ.zə ˈɔɪ.leɪ/

2 https://ftp.ncbi.nlm.nih.gov/pub/lu/MedCPT/pubmed_embeddings/

## References

[1] Cao Y, Liu F, Simpson P, Antieau L, Bennett A, Cimino JJ, Ely J, Yu H. AskHERMES: an online question answering system for complex clinical questions. J Biomed Inform. 2011;44(2):277–88. 21256977 10.1016/j.jbi.2011.01.004 PMC3433744

[2] Hristovski D, Dinevski D, Kastrin A, Rindflesch TC. Biomedical question answering using semantic relations. BMC Bioinform. 2015;16:1–14. 10.1186/s12859-014-0365-3 PMC4307891 25592675

[3] Mollá D, Vicedo JL. Question answering in restricted domains: an overview. Comput Linguist. 2007;33(1):41–61. 10.1162/coli.2007.33.1.41

[4] Ni Y, Zhu H, Cai P, Zhang L, Qui Z, Cao F. CliniQA: highly reliable clinical question answering system. In: Quality of life through quality of information. IOS Press; 2012. pp. 215–219. 22874183

[5] Vaswani A, Shazeer N, Parmar N, Uszkoreit J, Jones L, Gomez AN, Kaiser Ł, Polosukhin I. Attention is all you need. In: Advances in neural information processing systems; 2017. vol. 30.

[6] Devlin J, Chang M-W, Lee K, Toutanova K. Bert: Pre-training of deep bidirectional transformers for language understanding. arXiv preprint arXiv:181004805 2018.

[7] Radford A, Wu J, Child R, Luan D, Amodei D, Sutskever I. Language models are unsupervised multitask learners. OpenAI blog. 2019;1(8):9.

[8] Brown T, Mann B, Ryder N, Subbiah M, Kaplan JD, Dhariwal P, Neelakantan A, Shyam P, Sastry G, Askell A. Language models are few-shot learners. In: Advances in neural information processing systems; 2020. vol. 33, pp. 1877–1901.

[9] Achiam J, Adler S, Agarwal S, Ahmad L, Akkaya I, Aleman FL, Almeida D, Altenschmidt J, Altman S, Anadkat S. Gpt-4 technical report. arXiv preprint arXiv:230308774. 2023.

[10] Raffel C, Shazeer N, Roberts A, Lee K, Narang S, Matena M, Zhou Y, Li W, Liu PJ. Exploring the limits of transfer learning with a unified text-to-text transformer. J Mach Learn Res. 2020;21(140):1–67.34305477

[11] Introducing ChatGPT [https://openai.com/blog/chatgpt. Accessed 21 March 2024].

[12] Perplexity.ai [https://en.wikipedia.org/w/index.php?title=Perplexity.ai&oldid=1214662444#cite_note-5. Accessed 21 March 2024].

[13] Jiang Z, Xu FF, Araki J, Neubig G. How can we know what language models know? Trans Assoc Comput Linguist. 2020;8:423–38. 10.1162/tacl_a_00324

[14] Kojima T, Gu SS, Reid M, Matsuo Y, Iwasawa Y. Large language models are zero-shot reasoners. In: Advances in neural information processing systems; 2022. vol. 35, pp. 22199–22213.

[15] Reynolds L, McDonell K. Prompt programming for large language models: Beyond the few-shot paradigm. In: 2021; 2021. pp. 1–7.

[16] Lewis P, Perez E, Piktus A, Petroni F, Karpukhin V, Goyal N, Küttler H, Lewis M, Yih W-t, Rocktäschel T. Retrieval-augmented generation for knowledge-intensive nlp tasks. In: Advances in neural information processing systems; 2020. vol. 33, pp. 9459–9474.

[17] Izacard G, Grave E. Leveraging passage retrieval with generative models for open domain question answering. arXiv preprint arXiv:200701282. 2020.

[18] Lazaridou A, Gribovskaya E, Stokowiec W, Grigorev N. Internet-augmented language models through few-shot prompting for open-domain question answering. arXiv preprint arXiv:220305115. 2022.

[19] Siriwardhana S, Weerasekera R, Wen E, Kaluarachchi T, Rana R, Nanayakkara S. Improving the domain adaptation of retrieval augmented generation (RAG) models for open domain question answering. Transactions of the Association for Computational Linguistics. 2023;11:1–17. 10.1162/tacl_a_00530

[20] Xiong G, Jin Q, Lu Z, Zhang A. Benchmarking retrieval-augmented generation for medicine. arXiv preprint arXiv:240213178. 2024.

[21] Zakka C, Shad R, Chaurasia A, Dalal AR, Kim JL, Moor M, Fong R, Phillips C, Alexander K, Ashley E. Almanac—retrieval-augmented language models for clinical medicine. NEJM AI. 2024;1(2):Aloa2300068. 10.1056/AIoa2300068 PMC10857783 38343631

[22] Recursively split by character [https://python.langchain.com/v0.1/docs/modules/data_connection/document_transformers/recursive_text_splitter/. Accessed 12 July 2024].

[23] Mikolov T, Chen K, Corrado G, Dean J. Efficient estimation of word representations in vector space. arXiv preprint arXiv:13013781. 2013.

[24] Pennington J, Socher R, Manning CD. Glove: global vectors for word representation. In*: *2014; 2014. 1532–1543.

[25] New and improved embedding model [https://openai.com/blog/new-and-improved-embedding-model. Accessed 22 March 2024].

[26] Neelakantan A, Xu T, Puri R, Radford A, Han JM, Tworek J, Yuan Q, Tezak N, Kim JW, Hallacy C. Text and code embeddings by contrastive pre-training. arXiv preprint arXiv:220110005. 2022.

[27] Lee J, Yoon W, Kim S, Kim D, Kim S, So CH, Kang J. BioBERT: a pre-trained biomedical language representation model for biomedical text mining. Bioinformatics. 2019;36(4):1234–40. 10.1093/bioinformatics/btz682 PMC7703786 31501885

[28] Luo R, Sun L, Xia Y, Qin T, Zhang S, Poon H, Liu T-Y. BioGPT: generative pre-trained transformer for biomedical text generation and mining. Brief Bioinform. 2022;23(6):bbac409. 36156661 10.1093/bib/bbac409

[29] Jin Q, Kim W, Chen Q, Comeau DC, Yeganova L, Wilbur WJ, Lu Z. MedCPT: Contrastive Pre-trained Transformers with large-scale PubMed search logs for zero-shot biomedical information retrieval. Bioinformatics. 2023;39(11):btad651. 37930897 10.1093/bioinformatics/btad651 PMC10627406

[30] Pinecone overview [https://docs.pinecone.io/guides/getting-started/overview. Accessed 22 March 2024].

[31] Manning CD, Raghavan P, Schütze H. Introduction to information retrieval. Cambridge: Cambridge University Press; 2008.

[32] Mikolov T, Sutskever I, Chen K, Corrado GS, Dean J. Distributed representations of words and phrases and their compositionality. In: Advances in neural information processing systems; 2013. vol. 26.

[33] Robertson S, Zaragoza H, Taylor M. Simple BM25 extension to multiple weighted fields. In: Proceedings of the thirteenth ACM international conference on Information and knowledge management; 2004. pp. 42–49.

[34] A Python implementation of the BM25 ranking function. [https://github.com/nhirakawa/BM25, Accessed 12 July 2024].

[35] Elasticsearch [https://www.elastic.co/elasticsearch. Accessed 12 July 2024].

[36] Alsentzer E, Murphy JR, Boag W, Weng W-H, Jin D, Naumann T, McDermott M. Publicly available clinical BERT embeddings. arXiv preprint arXiv:190403323. 2019.

[37] Beltagy I, Lo K, Cohan A. SciBERT: A pretrained language model for scientific text. arXiv preprint arXiv:190310676. 2019

[38] Chiu B, Crichton G, Korhonen A, Pyysalo S. How to train good word embeddings for biomedical NLP. In: 2016; 2016. pp. 166–174.

