# Supplementary material for: Optimizing biomedical information retrieval with a keyword frequency-driven prompt enhancement strategy
Source: BMC Bioinformatics. 2024 Aug 27;25:281. doi: 10.1186/s12859-024-05902-7 (PMC11351623; doi:10.1186/s12859-024-05902-7)
Supplement: Supplementary file 2 — Additional file 2. Step-by-step instructions for setting up WeiseEule as a localhost application on user machine. [file 12859_2024_5902_MOESM2_ESM.pdf]

How to make the WeiseEule app run on Linux/Mac?

This document provides step-by-step instructions for setting up WeiseEule as a localhost application on your machine. The source code along with this guide is available in the following GitHub repository: https://github.com/wasimaftab/WeiseEule-LocalHost. As the app continues to evolve, the guidelines may change; therefore, users are encouraged to check the GitHub repo for up-to-date instructions. If any step is not clear or does not yield desired result, please report these in the issue section of the repository. We are committed to addressing any issues promptly.

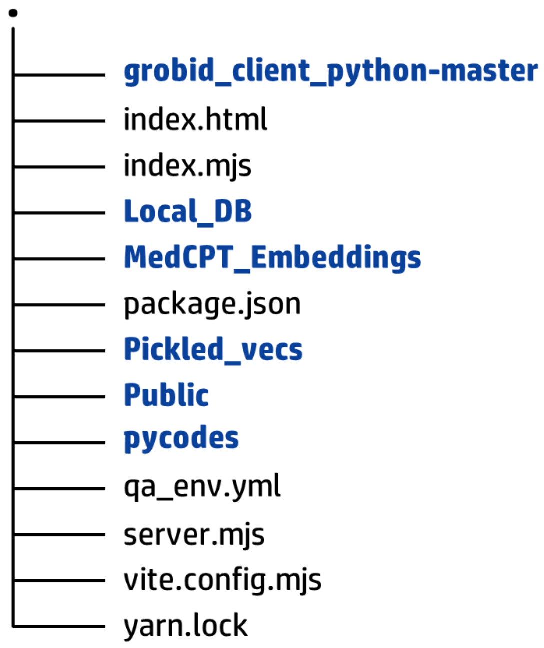

Fig. S1: App directory structure – The WeiseEule app directory contains the source code of the app and software documentation to set it up. Directories are shown in bold blue colored font.

1. Setting up JavaScript (JS) environment

<u>For Linux:</u>

Install `yarn` package manager by running the following commands

- Update package list by running the following command to ensure the most recent versions of packages and their dependencies are downloaded:

```
sudo apt update
```

- Install Node.js. If Node.js is not installed, then install it by running:

```
sudo apt install nodejs npm
```

- Install Yarn using npm (Node Package Manager) by running:

```
npm install --global yarn
```

- Verify the installation by checking the version of Yarn installed:

```
yarn --version
```

<u>For Mac:</u>

- Install Node.js if not installed by following the instructions in this link: https://treehouse.github.io/installation-guides/mac/node-mac.html
- Install Yarn using by following the instructions in this link: https://tecadmin.net/install-yarn-macos/

2. Setting up Python environment

- Install Conda by following instructions given in the official Anaconda documentation https://docs.anaconda.com/free/anaconda/install/linux/

- Mac user follow the instructions given in this link: https://docs.anaconda.com/anaconda/install/mac-os/

- Go to the app directory as given in Fig. S1 and create Python environment by running `conda env create -f qa_env.yml`

- After creating the new Python environment, activate it as follows:

```
conda activate qa_env
```

3. Get the API keys

- Obtain ENTREZ API KEY by following instructions given in the NCBI E-utilities page https://ncbiinsights.ncbi.nlm.nih.gov/2017/11/02/new-api-keys-for-the-e-utilities/

- Obtain PINECONE API KEY by following the guidelines from Fig. S2 (below).

- Get an OpenAI API KEY by following these steps https://maisieai.com/help/how-to-get-an-openai-api-key-for-chatgpt

4. Setting up API keys

Open `.bashrc` file located at your home directory and create environment variable to hold API keys as shown below:

```
export OPENAI_API_KEY='your-openai-key-here'

export ENTREZ_API_KEY='your-entrez-key-here'

export PINECONE_API_KEY='your-pinecone-key-here'
```

Note, keep the names of the environment variables for API keys as mentioned in this doc. If you would like to change the names, then feel free to edit the WeiseEule source code where they appear.

5. Setting up PINECONE environment

- Login to your account by registering at https://www.pinecone.io/ see Fig. S2 for more details.
- Open .bashrc file located in your home directory and set the pinecone index, region variables as shown below

```
export PINECONE_INDEX="your-pinecone-index-name"

export PINECONE_REGION="your-pinecone-region-name" (optional)
```

The index name and region are the one which you set during registration and index creation in https://pinecone.io

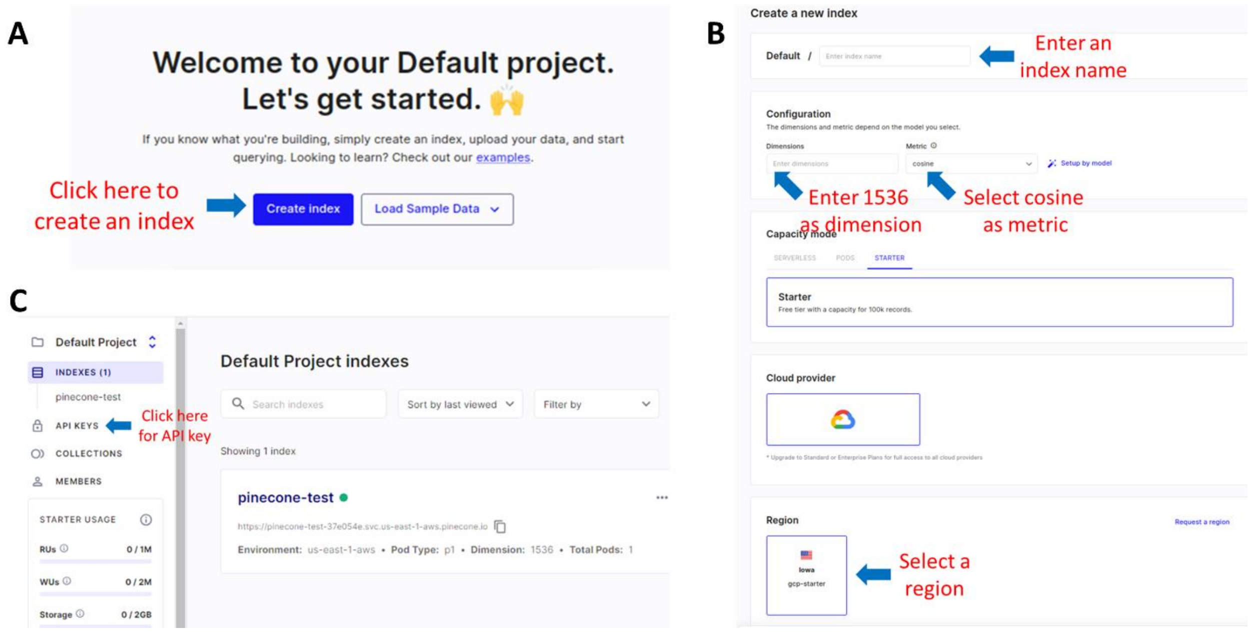

Fig. S2: Pinecone index creation – Register with https://pinecone.io to set up vector DB **(A)** Open an index creation wizard by clicking on the button as indicated by an arrow **(B)** Then populate

necessary fields in the wizard to create an index where namespaces will be stored. Select the free starter plan. **(C)** Obtain the API key by clicking on the button as indicated by an arrow.

6. Launching the app

First, make sure to activate the new python environment in a terminal as described in section 2 Setting up Python environment. Then using the same terminal go to the app directory and install the required JS packages locally by running following command: `yarn install`

After the successful execution of the above command a folder named `node_modules` will be created inside the app directory. Then you can launch the app by running the following command: `yarn start:both`

This will start the app on http://localhost:9000/

7. How to use WeiseEule app?

The following three steps are required to get answers to your queries as demonstrated in Fig. S3.

a. Select an LLM from the sidebar under `Set Parameters` navigation bar (Fig. S3, red circle 1) .

b. Select appropriate namespace under `Set Parameters` navigation bar (Fig. S3, red circle 2).

c. Type your question in the chat box on the main window and click the paper-plane icon or hit enter (Fig. S3, red circle 3). If you are unsure about certain parameters just hover your cursor on ? symbol which serve as help-point, will pop up an explanatory window.

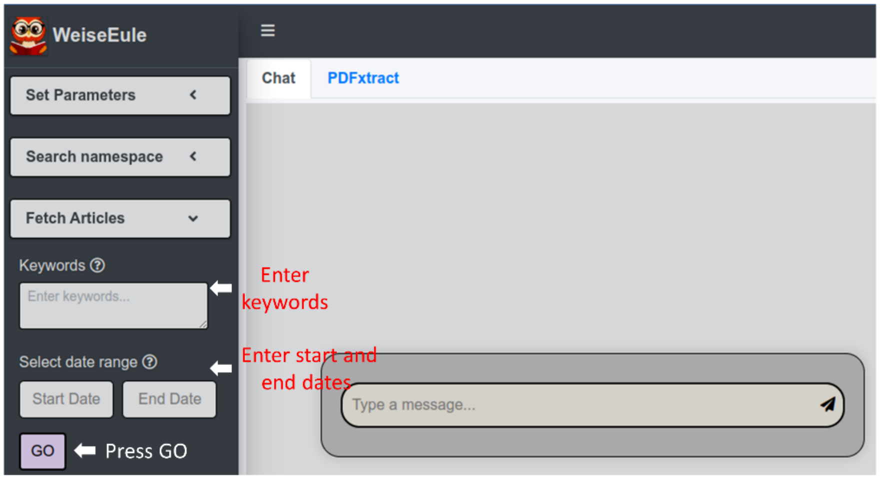

Fig. S3: Three essential steps to get answers – Parameters can be set by interacting with the input boxes and menus on the sidebar. Users should type their question in the chat box provided in the main window.

7.1 Chat response

By default, the app streams the answers to queries in PES1 mode using MedCPT retriever, to use the app in the PES2 mode, users need to set `Rerank` flag to TRUE (See Fig. S4). By default, the construction of prompts for `GPT-3.5` and `GPT-4` typically involves considering the top five and top ten rows/chunks, respectively. You can change this and set advanced params to configure the app for more sophisticated searches by clicking on the *Set Params* button and launching a modal with advance params (see Fig. S4). Hover the mouse on the question mark symbols (help-points) to obtain information about each param and, set accordingly. Note, that this step is optional and that, most of the time, there is no need to change params on this modal.

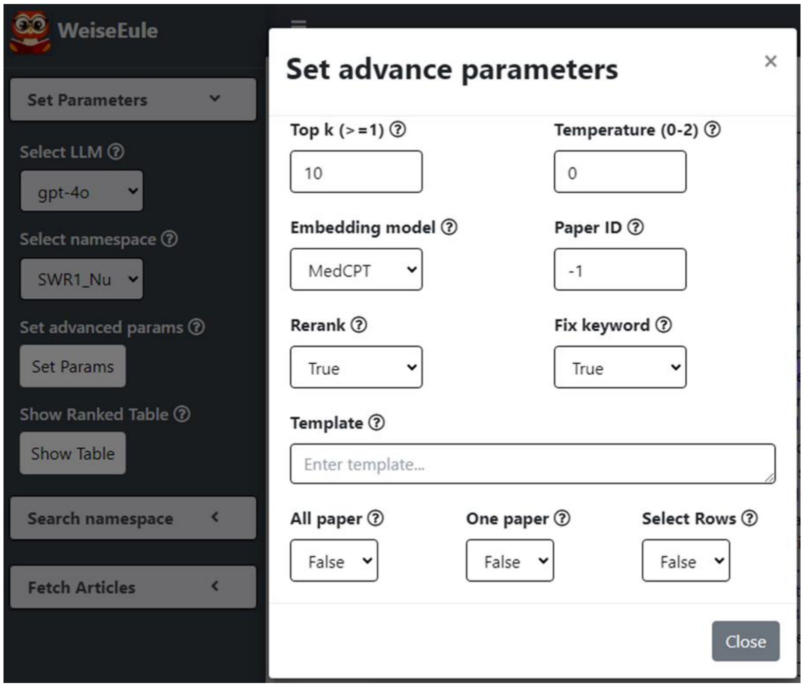

Fig. S4: Advance parameters – Use the `Set Params` button to launch a modal with advance parameters to configure the app for more sophisticated searches.

The chat response contains 3 parts: answer, reference and context as depicted in Fig. S5.

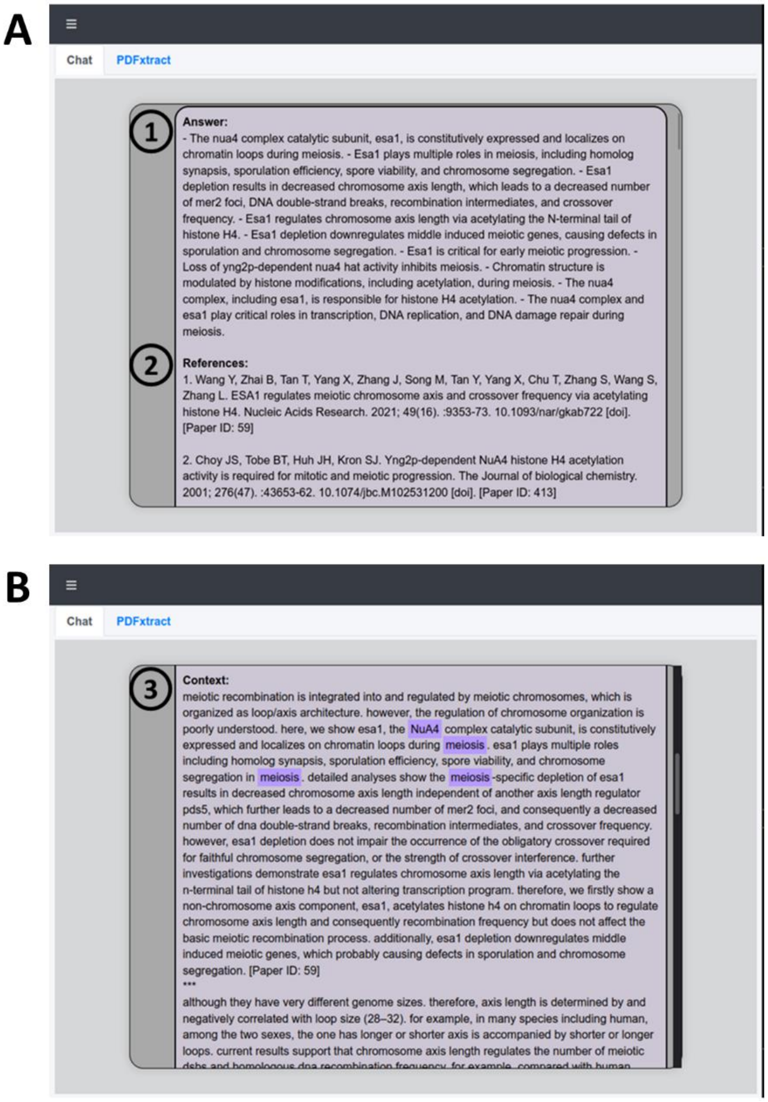

Fig. S5: Illustration of a chat response composed of three segments – (A) The answer (1) is followed by references (2), each marked with its respective publication ID. **(B)** Below the references lies the context (3), where keywords are highlighted, and each context section is identified by the corresponding publication ID. Contexts are separated from one another by the '***' symbol.

In PES2 mode, which ranks the relevant chunks based on the keyword frequencies, by default the keywords are extracted from the query automatically. However, sometimes the automatic approach

may not select all the desired keywords and may be preferable to extract them manually. If that is the case, then start the query with a `'#'` symbol and mark keywords using `**` as shown in Fig. S3. The idea here is to use the marked keywords from the user's query and rank paragraphs/chunks (from research articles) based on the keyword frequency. The rationale is that the chunks with higher frequency of user's keyword are more likely to have contexts useful to produce a good quality answer. The idea is depicted in the main text Figure 5. In the app, you can view the keyword frequency table by clicking on the `Show Table` button (See Fig. S6).

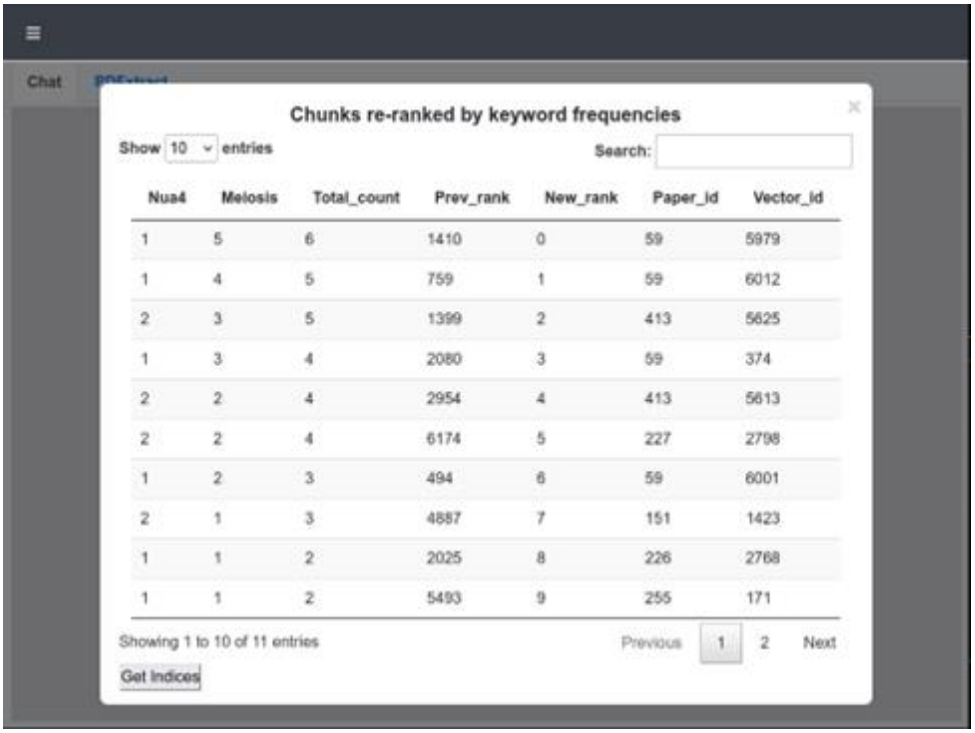

Nua4	Meiosis	Total_count	Prev_rank	New_rank	Paper_id	Vector_id
1	5	6	1410	0	59	5979
1	4	5	759	1	59	6012
2	3	5	1399	2	413	5625
1	3	4	2080	3	59	374
2	2	4	2954	4	413	5613
2	2	4	6174	5	227	2798
1	2	3	494	6	59	6001
2	1	3	4887	7	151	1423
1	1	2	2025	8	226	2768
1	1	2	5493	9	255	171

Fig. S6: Chunks are re-ranked by keyword frequencies – Each row refers to a chunk. The frequencies of keywords in each chunk, previous rank and the rank after frequency-based re-ranking is also shown.

7.2 Accessible LLMs

Currently we provide access to the four LLMs below, but advanced users can add or remove LLMs by modifying the source code:

1. `gpt-4`: This is `GPT-4` model and often provides better responses than GPT-3.5 models. It also costs more.
2. `gpt-4-1106-preview`: This is a variant of `GPT-4` family and performance wise comparable to `` `gpt-4` `` but slightly cheaper than that model.
3. `gpt-4o`: This is also `GPT-4` series model but it the fastest and most affordable flagship model from OpenAI. This model is selected by default.
4. `gpt-3.5-turbo`: This is `GPT-3.5` model, cost-effective but not precise for complex queries.
5. `gpt-3.5-turbo-1106`: This is an updated variant of `GPT-3.5` and may show slightly improved performance over `` `gpt-3.5-turbo` ``.

We recommend using `` `gpt-4o` ``, for complex queries seeking specific information.

7.3Look-up publications in namespace

It is also possible to search whether a publication is present in a selected namespace, as shown in Fig. S7. The search result not only reveals the publication's presence/absence but it also indicates whether the full text or just the abstract is accessible for the specified PubMed ID in that namespace. This is useful when judging the quality of a response or investigating why a specific publication was not included in the reference.

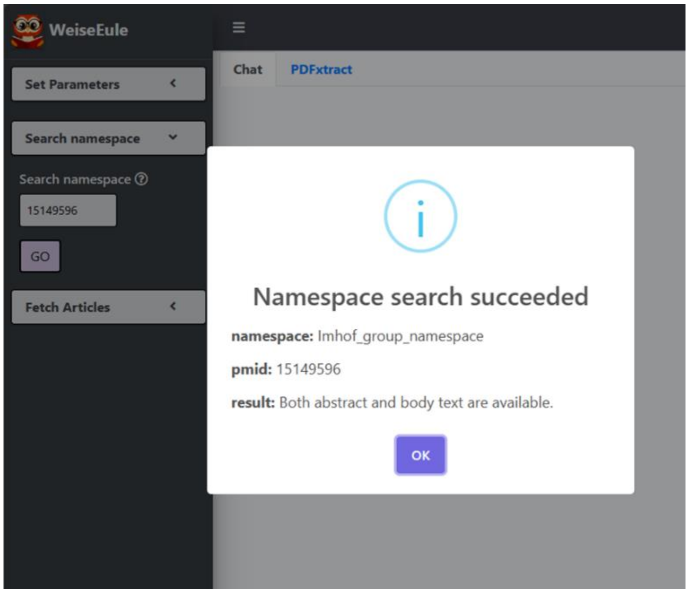

Fig. S7: Illustrates the search namespace feature – Input a valid PubMed ID and hit *GO* button.

7.4Exception/Error messages

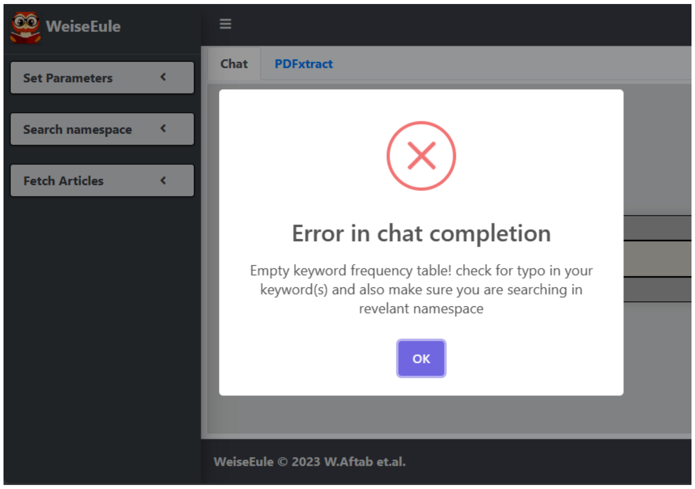

Fig. S8: Error message when chat completion fails.

Error messages come with an explanation. For example, when you see a message as shown in Fig. S8, it means that the keywords could not be found in any of the chunks in your namespace. There could be several reasons:

a. You have selected a namespace that is not relevant to your query.

b. There are typos in your keywords.

c. Or sometimes you have a paper in mind which you expect a specific publication to be retrieved to show up, but that it may not be part of your namespace. In that case, it is recommended to confirm if that the paper is indeed part of the selected namespace by using the 'search namespace' feature of the app (See Fig. S8). Sometimes only the abstract will be available and it is possible that the keywords are not mentioned there.

8. Namespace creation using GUI

Using the WeiseEule GUI, articles can be obtained from PubMed, as illustrated in Fig. S9. The namespace "name" is determined based on the entered keyword(s) to make the naming relevant. For example, if the entered keyword is "dosage compensation," and the text embedder model is "MedCPT" then the namespace is named as "dosage_compensation_MedCPT," where "MedCPT" part at the end implies that in this namespace, article chunks are embedded using MedCPT article encoder. In the future, we will provide separate fields on the GUI to change the chunk size and naming a namespace. Currently, you can achieve that by modifying the `chunk_size` and `namespace` variables in the endpoint: `@app.websocket("/ws/fetch_articles")` located in `pycodes/ fastapi_app.py` inside the app directory (see Fig. S1).

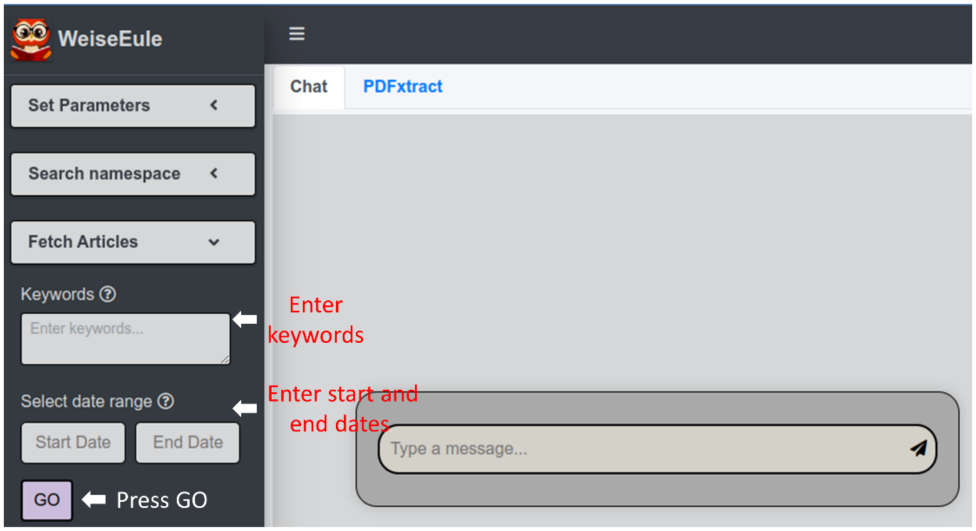

Fig. S9: Article accumulation – Only publications that contain keywords either on the title or abstract are considered for fetching.

Note, this approach can create a namespace by fetching the full text of articles that are available (in xml format) on the PMC server, and for others, it only fetches the abstract from PubMed. However, for many articles in PMC the full text is not available on the PMC in xml format. In those cases, we have a python script called `PDF2NAMEPSACE_with_PMIDs.py` to download those articles as PDFs and then extract texts to augment the existing namespace. It may be run either in a terminal or in an IDE such as `Spyder`. Note, the PDFs are saved in a local folder pdf_downloaded inside the app directory.

9. Namespace creation for custom PDFs

In some cases, a user may already have a collection of PDFs in a local folder that can serve as a knowledge base. In that case, namespace creation is only possible by running the `PDF2NAMEPSACE_NO_PMIDs.py` file either in a terminal or in an IDE such as `Spyder`. In the

future, we will also support this feature via GUI. Once the namespace is generated querying it can be done as explained earlier (see Fig. S3).

10.Downloading all the metadata from vector DB for PES2

It is important to note that in order to use PES2, one must download all the metadata from the corresponding namespace in the vector DB. To do that run the following python code provided in the app directory: `pycodes/extract_all_meta_pinecone.py` either in a terminal or in an IDE such as `Spyder`. The code will prompt you to enter a namespace and you must enter a valid namespace hosted in the pinecone server. Without this step, PES2 will not work.

11.Parameter settings used to evaluate PES1

To configure the WeiseEule app to work in PES1 mode, first select `gpt-4o` as LLM. Then launch the *Advance parameters* modal (See sub-section 7.1 and Fig. S4) and set the `Rerank` and `Top k` parameters as `False` and `10` respectively as shown in Fig. S10.

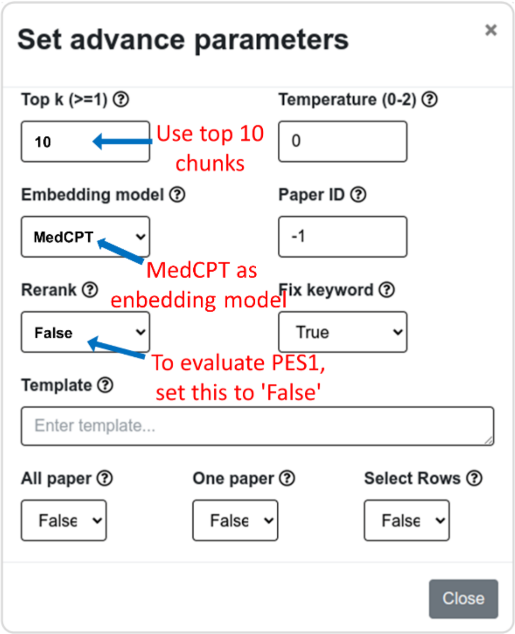

Fig. S10: Parameter settings used to evaluate PES1 – Launch the `Set Params` modal and set the `Rerank` and `Top k` parameters to `False` and `10`, respectively.

12.Parameter settings used to evaluate PES2

To configure the WeiseEule app to work in PES2 mode, you must first select `gpt-4o` as the LLM. Then set the `Rerank` parameter to `True` as shown in Fig. S11. By default, top 10 chunks is selected as context to answer the query.

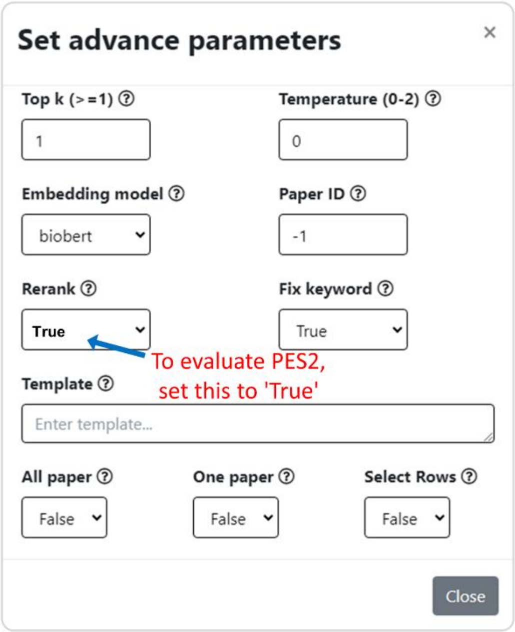

Fig. S11: Parameter settings used to evaluate PES2 – Launch the `Set Params` modal and set the `Rerank` parameter to `True`.

13.Downloading MedCPT embeddings

To use the *Find relevant articles* feature, you need to download MedCPT embeddings for PubMed articles from this link: https://ftp.ncbi.nlm.nih.gov/pub/lu/MedCPT/pubmed_embeddings/. The embeddings generated by the MedCPT article encoder are organized as follows:

The embeddings files are divided into chunks, with each chunk containing approximately 1 million articles. For instance, chunk 0 includes PMIDs ranging from 0 to 999,999. Due to some articles being missing, the exact number of articles per chunk may vary. Each chunk comprises the following three files:

a. `embeds_chunk_{chunk_id}.npy` is a numpy array with the shape (N, 768), where N is roughly 1 million and 768 is the embedding dimension.

b. `pmids_chunk_{chunk_id}.json` is a list of N PMIDs corresponding to the vectors.

c. `pubmed_chunk_{chunk_id}.json` is a dictionary mapping these N PMIDs to their actual contents.

Suppose you want to search inside chunk '36', then you must download the above three files corresponding to *chunk_id* 36. Missing any will pop-up an error message as shown in Fig. 12.

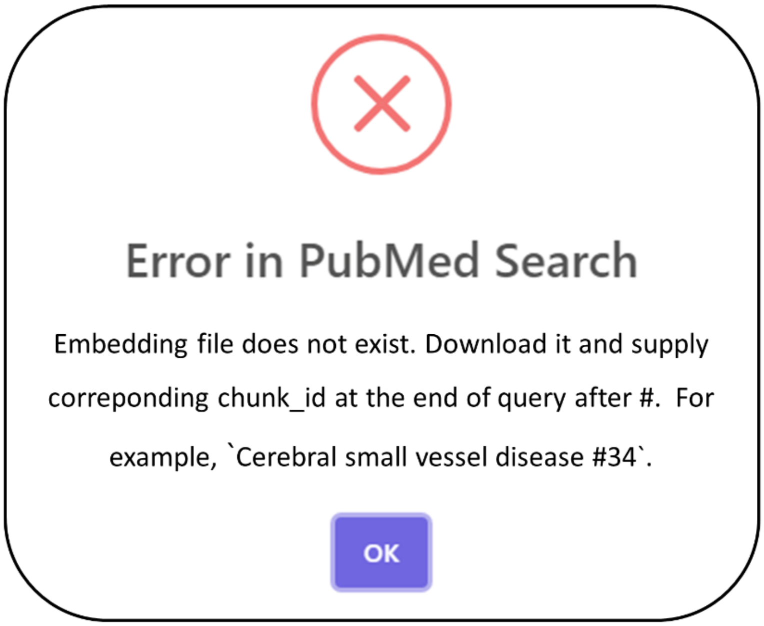

Fig. S12: Error in PubMed search – This figure illustrates the error encountered when any of the following files are missing: *embeds_chunk_{chunk_id}.npy*, *pmids_chunk_{chunk_id}.json*, or

pubmed_chunk_{chunk_id}.json. Specifically, it shows the error that occurs when the *embeds_chunk_{chunk_id}.npy* file is missing.

Note:

1. Guidelines in this documentation were tested on Ubuntu 22.04 Linux OS, and it should also work on Mac OS. Windows users can also set up the app environment by following these guidelines with some adjustments, particularly during installation of packages and exporting environment variables as these processes differs between Windows and Linux/Mac systems.
2. In the future, we will add documentation on how to set up WeiseEule on Windows. For future updates, users are requested to check the GitHub repository. Additionally, users are welcome to contribute by requesting features or by submitting issues on the GitHub page.
